# Diabetes and obesity reduce FIB-4 accuracy in MASLD referral pathways

**DOI:** 10.1016/j.jhepr.2026.101735

**Published:** 2026-01-12

**Authors:** Abdel-Aziz Shaheen, Elizabeth Baguley, Mark G. Swain, Matthew Tam, Mang Ming Ma, Giada Sebastiani, Jason Jiang, Frank Lee, Alexandra Medellin, Juan G. Abraldes

**Affiliations:** 1 Division of Gastroenterology and Hepatology, Department of Medicine, Cumming School of Medicine, University of Calgary, Calgary, Alberta, Canada; 2 Department of Community Health Sciences, Cumming School of Medicine, University of Calgary, Calgary, Alberta, Canada; 3 Division of Gastroenterology, Liver Unit, University of Alberta, Edmonton, Alberta, Canada; 4 Division of Gastroenterology and Hepatology, McGill University, Montreal, Quebec, Canada; 5 Provincial Research Data Services, Health Shared Services, Calgary, Alberta, Canada; 6 Department of Radiology, Cumming School of Medicine, University of Calgary, Calgary, Alberta, Canada

**Keywords:** MASLD, Metabolic dysfunction-associated steatotic liver disease, risk-stratification, primary care, advanced fibrosis

## Abstract

**Background & Aims:**

Primary care referral pathways often use FIB-4 to triage metabolic dysfunction-associated steatotic liver disease (MASLD), but its accuracy may vary by patient characteristics. We aimed to evaluate FIB-4 performance against elastography, compare it with other non-invasive tests, assess effect modification by BMI, diabetes, sex, ALT, and age, and calibrate 2D shear-wave elastography (2D-SWE) and vibration-controlled transient elastography (VCTE) thresholds.

**Methods:**

We analyzed two cohorts with paired serum scores and elastography: Calgary (2D-SWE; N = 8,126) and Edmonton (VCTE; N = 985). We summarized fibrosis-risk distributions and used exceedance probabilities to align 2D-SWE with guideline-recommended VCTE cut-offs.

**Results:**

In the Calgary cohort, median 2D-SWE was 4.6 kPa (IQR 3.9–5.8) and 9% had liver stiffness ≥8 kPa; FIB-4 was <1.30 in 70% and ≥2.67 in 5%. In the Edmonton cohort, median VCTE was 5.3 kPa (4.4–6.6) and 14% had liver stiffness ≥8 kPa; FIB-4 was <1.30 in 77% and ≥2.67 in 3%. In exceedance models, at a FIB-4 of 1.30, the probability of liver stiffness ≥8 kPa was significantly higher in individuals with diabetes or BMI ≥30 kg/m^2^ than in those without these risk factors. Regression analyses showed that BMI and diabetes materially increased the probability of liver stiffness ≥8 kPa at a given FIB-4, whereas sex, elevated ALT, and age had smaller effects. Using the recommended 2D-SWE thresholds of 9, 13, 17 kPa, we observed an expected stepwise correspondence with VCTE 15, 20, 25 kPa, supporting harmonised elastography cut-offs across modalities.

**Conclusion:**

FIB-4 alone under-triages patients with diabetes and/or obesity. These patients should be fast-tracked to elastography even when FIB-4 is <1.30. We recommend validating these findings in other cohorts, as they may affect triaging practices.

**Impact and implications:**

Recent guidelines recommend clinical care pathways for risk stratification of patients with MASLD (metabolic dysfunction-associated steatotic liver disease) using sensitive serum-based markers (*e.g*. fibrosis 4 index [FIB-4]) as a first step, with a cut-off of <1.30 to rule out advanced fibrosis. In patients with diabetes or obesity, the interpretation of a FIB-4 threshold of <1.30 is different than in patients without these conditions. This finding suggests the need to refine referral pathways in which FIB-4 is used as a first test. New referral models incorporating patient characteristics could improve risk stratification of patients with MASLD who need specialized liver care.

## Highlights

• In patients with MASLD, FIB-4 under-triages those with diabetes or obesity.
• Diabetes/BMI ≥30 increase the risk of ≥8 kPa stiffness even at FIB-4 <1.30.
• BMI and diabetes strongly modify FIB-4 performance; sex, ALT, and age have smaller effects.
• 2D-SWE thresholds align with VCTE cut-offs, supporting harmonized elastography use.

## Graphical abstract

**Figure:**
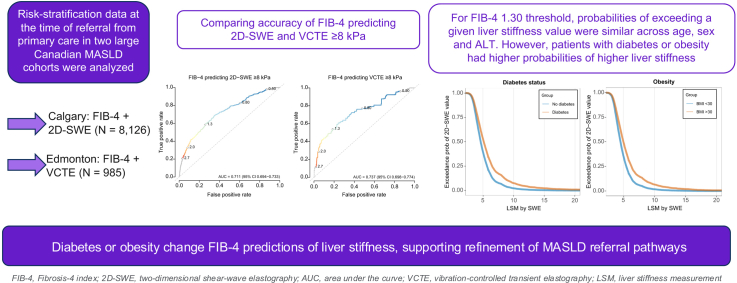

## Introduction

The prevalence of metabolic dysfunction-associated steatotic liver disease (MASLD) has significantly increased over the last two decades, with more than 30% of the population worldwide reported to be affected.^1^^,^^2^ Patients with MASLD and advanced liver fibrosis are more likely to have liver-related causes of morbidity and mortality compared to those with lesser degrees of fibrosis.^3^ Moreover, advanced liver fibrosis is associated with higher cardiovascular disease-related morbidity and mortality.^4^

Recent guidelines recommend clinical care pathways for risk-stratification of patients with MASLD using serum-based markers (*e.g.* fibrosis-4 index [FIB-4]) with high negative predictive value (NPV) cut-offs as a first step.^5^^,^^6^ The main goal of MASLD clinical pathways is to use simple serum-based markers first, followed by radiological modalities (*e.g*. vibration-controlled transient elastography [VCTE] or two-dimensional shear wave elastography [2D-SWE]) or specific serum-based markers (such as enhanced liver fibrosis [ELF]) to identify patients at-risk of advanced fibrosis who may benefit from further evaluation, management and treatment.^5^^,^^7^^,^^8^ Indeed, guidelines for the use of the recently approved treatment for MASLD (resmetirom) propose elastography-based decision rules to initiate treatment, making elastography the new standard in clinical practice to risk stratify patients with MASLD.^9^^,^^10^

As FIB-4 values increase with older age, it is recommended to use different FIB-4 cut-offs according to age, with a FIB-4 cut-off of ≥1.3 for patients aged <65 years and ≥2.0 for those aged ≥65 years.^11^ While patients with diabetes mellitus have a higher prevalence of advanced fibrosis, current recommendations are to use the same FIB-4 cut-offs for hepatology referral, and second-line assessment with liver stiffness measurement (LSM) regardless of diabetes status.^5^^,^^11^ Previous studies showed poor accuracy of FIB-4 to identify advanced fibrosis among patients with diabetes, and FIB-4 accuracy was dependent on BMI among those patients.^12^^,^^13^ Therefore, in this study, we used data from two large cohorts of patients with MASLD who were initially evaluated in primary care and underwent risk stratification with both FIB-4 and LSM (by 2D-SWE or VCTE) to understand the impact of patient characteristics on the association between FIB-4 and LSM. Our primary objective was to evaluate the performance of FIB-4 as an initial risk stratification tool in a large primary care cohort of patients with risk factors for MASLD, considering key clinical factors such as age, sex, liver enzymes, BMI, and diabetes mellitus, using 2D-SWE or VCTE as reference tests. Our secondary objective was to develop a predictive model integrating FIB-4 and these clinical variables to estimate different LSM thresholds with potential therapeutic relevance.

## Patients and methods

### Study setting and design

In this retrospective study, we included participants evaluated through the first phase of Calgary MASLD pathway implementation between 2017 and 2022; and the Edmonton MASLD pathway between 2016 and 2021. The development and performance of the Calgary and Edmonton clinical care pathways have been previously published.^14^^,^^15^ The Calgary pathway serves primary care providers (PCPs) in the Calgary Health Zone (population of ∼1.6 million) providing them with a framework to directly assess patients with a history of metabolic syndrome (*e.g*. diabetes, dyslipidemia or obesity) or evidence of steatotic liver on prior imaging for 2D-SWE assessment provided through community-based radiology providers. PCPs were expected to complete a full workup to rule out other causes of chronic liver disease, such as metabolic dysfunction- and alcohol-related liver disease (MetALD) and viral hepatitis, by following a pathway-outlined stepwise algorithm. 2D-SWE was chosen as the first step in MASLD assessment because, in preliminary discussions about pathway design and implementation, PCPs preferred 2D-SWE over FIB-4. This preference was due to a simpler referral process, reduced reliance on calculating FIB-4 in busy primary care settings, and the availability of radiology providers using a single validated 2D-SWE platform (Aplio i800; Canon Medical Systems).^14^^,^^16^ Patients with 2D-SWE ≥8.0 kPa, or inconclusive results, were referred to a single MASLD hepatology clinic in Calgary. The Edmonton primary care MASLD pathway had different referral criteria: abnormal transaminases in the context of metabolic syndrome or a finding of steatosis on prior imaging. During the period between 2016 and 2021 all patients had FIB-4 and VCTE regardless of FIB-4 values.

### Study population

During the study period, 12,122 adult individuals were assessed by 2D-SWE in the Calgary health zone by primary care. As PCPs did not consistently order aspartate aminotransferase (AST) to calculate FIB-4 at the time of 2D-SWE assessment, 8,590 patients had an available AST value to calculate FIB-4 within 6 months of 2D-SWE assessment. Patients excluded from the study cohort included those consuming more than 10 standard alcoholic drinks per week for women, or 15 standard drinks per week for men (n = 21), and pediatric patients (n = 1). Consistent with current nomenclature, our cohort includes patients with MASLD and MetALD. Patients with inconclusive 2D-SWE (n = 374) were excluded. Therefore, 8,126 patients were included in this study. Similar exclusion criteria were implemented in the Edmonton cohort and there were 985 patients eligible for this study with available FIB-4 and valid VCTE. Inclusion and exclusion criteria for our MASLD cohorts are shown in Fig. S1.

### Data sources

We used the Calgary and Edmonton MASLD pathway databases, which include 2D-SWE and VCTE radiology data; the Alberta provincial laboratory database, capturing ≥97% of all laboratory investigations for Alberta residents; and Alberta administrative databases (inpatient, outpatient, and physician claims).

### Outcomes and main variables of interest

The study had two outcomes: (a) estimation of the probability of exceeding LSM thresholds by 2D-SWE or VCTE across FIB-4 values; and (b) assessment of the performance of FIB-4 in predicting LSM according to age, sex, BMI, transaminases, and diabetes status. The main variables of interest were: a) biological sex; b) diabetes mellitus defined as: Hemoglobin A1c (HbA1c) >6.4%, a diagnosis code of diabetes by a physician, or patients using diabetes-related medications and a history of HbA1c >6.4%; and c) BMI (weight in kg/m^2^). We also created two obesity measurements separating class 1 *vs*. class 2/3 (dichotomous BMI: <35 and ≥35) or living with obesity *vs*. not (BMI: <30 and >30) to understand the impact of obesity on study outcomes. We followed this classification as less than 5% of our cohort were of Asian ethnicity. We defined normal alanine aminotransferase (ALT) level at baseline as <30 U/L for men, and <25 U/L for women.^17^ We compared FIB-4 with other non-invasive tests (NITs) for fibrosis, including LiverPRO,^18^ and the fibrotic NASH index (FNI)^8^ scores against elastography-defined elevated stiffness: 2D-SWE ≥8 kPa (Calgary) and VCTE ≥8 kPa (Edmonton). Pairwise tests used pairwise complete cases. ROC/AUROC were estimated non-parametrically with bootstrap 95% CIs (100 replicates), and pairwise AUC differences were tested by DeLong (two-sided α = 0.05).^19^

### Covariates

Laboratory data were obtained at baseline (within 6 months of 2D-SWE assessment). Serum ALT, AST, albumin, alkaline phosphatase, gamma glutamyltransferase, platelets, triglycerides, high-density lipoprotein, low-density lipoprotein, total bilirubin, international normalized ratio, creatinine, and HbA1c were collected. We documented the presence of comorbidities using the Charlson comorbidity index^20^ defined using categorical variables (0, 1 or ≥2 comorbidities). Moderate or severe liver disease comorbidities as well as diabetes mellitus components were removed from the Charlson comorbidity index.

### Statistical analyses

Patient characteristics were described as median (IQR) or percentage (n) for the MASLD pathway primary care-based cohort. Chi squared (*X*^2^) test and Wilcoxon rank-sum test were used to study differences between our two study cohorts according to our main exposure variables. Cut-off points for LSM determined by 2D-SWE and used to define "high risk" MASLD are arbitrary, and there is ongoing discussion around the threshold of LSM by 2D-SWE that should lead to hepatology referral or to treatment initiation. To model the associations between FIB-4 and 2D-SWE, and the effect of the modifying variables, we used ordinal regression for a continuous outcome (in this case LSM by 2D-SWE) (with the orm function in the rms package, R software). In this way we avoided assuming beforehand a definite target threshold of 2D-SWE.^21^ This model allows calculation of exceedance probabilities for every potential 2D-SWE threshold, according to values of the predictors. Details of the modeling process and illustrative examples are provided in the supplementary methods.^22^

To establish an indirect comparison of VCTE and 2D-SWE values, we modeled the association between FIB-4 and VCTE with ordinal regression in the Edmonton sample, and between FIB-4 and 2D-SWE in a subsample of the Calgary cohort, matched 1:1 with the Edmonton cohort by age, sex, presence of diabetes, BMI and abnormal transaminases. We used nearest neighbor matching without replacement, with a 1:1 matching ratio to pair each participant in the Calgary cohort with the closest match in the Edmonton cohort based on a logistic regression score. Matching was performed with the MatchIt package in R, specifying method = "nearest" and distance = "logit" for the matching algorithm.^23^ Results of the matching are shown in the supplementary methods. This approach, by anchoring both cohorts on FIB-4, allows an indirect comparison of VCTE and 2D-SWE thresholds. To assess potential selection bias related to missing AST (and therefore missing FIB-4), we compared baseline demographics, laboratory values, comorbidities, and 2D-SWE between patients with and without an available FIB-4. We also conducted a sensitivity analysis using BMI ≥30 kg/m^2^ in addition to our primary stratification at BMI ≥35 kg/m^2^. Analyses were performed using Stata IC (version 17.1, Texas, USA) and R version 4.3.2 (R Core Team, Vienna, Austria). The study protocol was reviewed and approved by the Research Ethics Committee at the University of Calgary, Calgary, Alberta (REB17-2142).

## Results

### MASLD pathway cohort characteristics

Patient characteristics of the Calgary and Edmonton MASLD primary care cohorts are shown in Table 1. In the Calgary MASLD cohort, the median age was 54 years (IQR 43-63) and 53% of patients were female. Obesity (median BMI 31.2 [IQR 27.6-35.7]) and diabetes (34%) were the main indications for enrollment in the MASLD pathway by primary care. The median ALT was 38 U/L (IQR 25-60), with 5,720 (70%) patients in the Calgary cohort having an elevated ALT level. In contrast, patients in the Edmonton cohort were younger (median age 43 [35-53]), more likely to be male (67%), and most had elevated ALT and AST levels. Characteristics of patients with FIB-4 ≥1.30 or 2D-SWE ≥8 kPa in the Calgary cohort are shown in Table 1.

**Table 1:** Patient characteristics.

Characteristic	Calgary MASLD cohort N = 8,126	Calgary MASLD patients with FIB-4 ≥1.30 n = 2,476	Calgary MASLD patients with 2D-SWE ≥8 kPa n = 754	Edmonton MASLD cohort N = 985
Age, yrs.	54 (43-63)	63 (56-69)	60 (51-67)	43 (35-53)
Female sex	52.5% (4,261)	48.5% (1,199)	53.5% (402)	32.6% (321)
BMI (kg/m^2^)	31.2 (27.6-35.7)	30.9 (27.3-35.2)	34.6 (29.9-40.4)	30.9 (27.7-35.1)
Baseline investigations				
ALT, U/L	38 (25-60)	38 (25-61)	40 (26-66)	61 (42-87)
AST, U/L	28 (21-40)	38 (27-56)	36 (23-55)	36 (28-49)
Albumin, G/L	39 (37-41)	39 (37-41)	38 (36-40)	45 (43-47)
ALP, U/L	77 (64-95)	78 (64-99)	86 (69-108)	80 (67-97)
Platelets, 10E^9^/L	251 (210-297)	205 (172-241)	221 (176-273)	236 (199-278)
Triglycerides, mmol/L	1.8 (1.2-2.5)	1.6 (1.1-2.3)	1.7 (1.2-2.4)	1.7 (1.2-2.3)
HDL, mmol/L	1.2 (1.0-1.4)	1.2 (1.0-1.5)	1.1 (0.9-1.3)	1.1 (1.0-1.3)
LDL, mmol/L	2.6 (1.9-3.3)	2.3 (1.7-3.0)	2.2 (1.6-2.9)	2.9 (2.3-3.5)
Total bilirubin, μmol/L	8 (6-11)	9 (7-13)	9 (6-13)	11 (9-15)
HbA1c, %	5.8 (5.5-6.2)	5.8 (5.5-6.5)	6.1 (5.6-7.1)	5.7 (5.4-6.1)
Diabetes mellitus	34.1% (2,772)	42.5% (1,052)	57.6% (434)	22.1% (218)
Comorbidities, Charlson Index				
0	34.4% (2,791)	24.5% (607)	18.2% (137)	
1	21.7% (1,769)	19.5% (482)	15.0% (113)	
≥2	43.9% (3,566)	56.0% (1,387)	66.8% (504)	
2D-SWE or VCTE, valid measurements in kPa 2D-SWE ≥8 kPa	4.6 (3.8-5.7) 9.3% (754)	5.2 (4.2-6.7) 18.0% (442)		5.3 (4.4-6.6) 13.6% (134)
FIB-4	0.97 (0.67-1.42)		1.48 (0.98-2.42)	0.84 (0.61-1.26)
FIB-4 ≥1.30	30.4% (2,476)		58.6% (442)	23.5% (231)
FIB-4 ≥2.67	5.1% (414)		20.8% (157)	3.2% (31)

Data presented as median (IQR) or % (n). 2D-SWE, two-dimensional shear wave elastography; ALP, alkaline phosphatase; ALT, alanine aminotransferase; AST, aspartate aminotransferase; FIB-4, fibrosis 4 index; HbA1c, hemoglobin A1C; VCTE, vibration-controlled transient elastography.

Comparing Calgary patients with available FIB-4 data (N = 8,126) to those without (n = 3,532), age (54 *vs.* 55 years) and BMI (31.2 *vs.* 31.5 kg/m^2^) were similar. Patients with FIB-4 showed higher female representation (53% *vs.* 49%) and slightly higher diabetes prevalence (34% *vs.* 32%). Median 2D-SWE values were similar between groups (4.6 *vs*. 4.5 kPa), indicating no difference in disease severity by FIB-4 availability (Table S1).

### Performance of FIB-4 and other NITs compared to 2D-SWE and VCTE in the primary care MASLD cohorts

We first assessed the performance of FIB-4 in risk stratifying our MASLD cohorts using recommended cut-offs. In the Calgary MASLD cohort, FIB-4 classified 5% as ≥2.67, 25% between 1.30 and 2.66, and 70% <1.30. In the Edmonton MASLD cohort, FIB-4 classified 3% as ≥2.67, 20% between 1.30 and 2.66, and 77% <1.30. In the Calgary cohort the median 2D-SWE LSM value was 4.6 kPa (IQR: 3.8-5.7) and 9% had 2D-SWE ≥8.0 kPa. In the Edmonton cohort, the median VCTE LSM measurement was 5.3 kPa (IQR 4.4-6.6) and 14% had VCTE ≥8.0 kPa (Table 1).

**Fig. 1:**
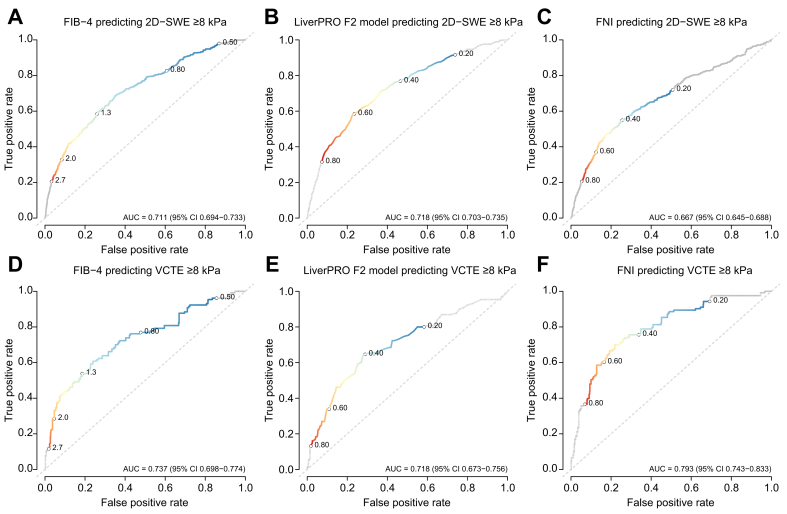
Receiver-operating characteristic curves showing the discriminative value of non-invasive tests for detecting 2D-SWE ≥8 or VCTE ≥8. Receiver-operating characteristic curves showing the discriminative value of FIB-4, LiverPRO and FNI for detecting 2D-SWE ≥8 (A-C) or VCTE ≥8 (D-F). 2D-SWE, two-dimensional shear wave elastography; FIB-4, fibrosis-4 index; FNI, fibrotic NASH index; VCTE, vibration-controlled transient elastography.

Prevalence of LSM ≥8.0 kPa measured by 2D-SWE in the Calgary MASLD cohort or VCTE in the Edmonton MASLD cohort among different categories of FIB-4 (<1.30, 1.30-2.66, and ≥2.67) are presented in Table 2. Furthermore, data on 2D-SWE ≥6.5 kPa are also presented. Overall, prevalence of 2D-SWE ≥8.0 kPa or VCTE ≥8.0 kPa was significantly higher among FIB-4 categories in patients with elevated ALT, diabetes, females, or BMI >35, *p* <0.001 (Table 2). However, the prevalence of 2D-SWE ≥8 kPa among patients with FIB-4 <1.30 was higher among patients with normal ALT compared to elevated ALT (7% *vs*. 5%, *p* = 0.013); 2D-SWE ≥8.0 kPa ratios were similar between males and females in patients with FIB-4 values between 1.30 and 2.67 (15% *vs*. 13%, *p* = 0.110); and VCTE ≥8.0 kPa was similar between patients with BMI <35 and BMI ≥35 in patients with FIB-4 values ≥2.67 (63% *vs.* 50%, *p* = 0.761). Findings were directionally consistent when obesity was defined as BMI ≥30 kg/m^2^; estimates and contrasts were similar (Table S5). Interestingly, ratios of 2D-SWE or VCTE LSM measurements ≥8.0 kPa were two times higher among patients with diabetes than in patients without diabetes in all FIB-4 categories. In Calgary, AUROCs were FIB-4 0.709 (95% CI 0.689–0.728), LiverPRO 0.718 (0.701–0.736), and FNI 0.669 (0.648–0.689). FIB-4 *vs*. LiverPRO *p =* 0.433, FIB-4 *vs*. FNI *p* <0.001, LiverPRO *vs*. FNI *p* <0.001. In Edmonton, AUROCs were FNI 0.793 (0.743–0.833), FIB-4 0.737 (0.698–0.774), and LiverPRO 0.718 (0.673–0.756). FNI was more accurate compared to FIB-4 *p =* 0.023 and LiverPRO *p =* 0.008. FIB-4 performed similarly to LiverPRO *p =* 0.372 (Fig. 1).

**Table 2:** Association between 2D-SWE, VCTE and FIB-4 per groups of interest in Calgary and Edmonton cohorts.

Groups of interest	FIB-4 categories	Calgary cohort 2D-SWE ≥8 kPa	Calgary cohort 2D-SWE ≥6.5 kPa	Edmonton cohort VCTE ≥8 kPa
Main cohort (Calgary = 8,126) (Edmonton = 985)	<1.30	5.5% (312/5,670)	10.7% (604/5,670)	8.1% (61/754)
	1.30-2.66	14.0% (285/2,042)	23.4% (477/2,042)	27.0% (54/200)
	≥2.67	37.9% (157/414)	47.8% (198/414)	61.3% (19/31)
Patients with elevated ALT (Calgary = 5,720) (Edmonton = 907)	<1.30	5.0% (200/3,997)	10.6% (422/3,997)	8.6% (60/702)
	1.30-2.66	15.6% (217/1,394)	25.8% (360/1,394)	28.8% (51/177)
	≥2.67	40.7% (134/329)	51.1% (168/329)	64.3% (18/28)
Patients with normal ALT (Calgary = 2,406) (Edmonton = 78)	<1.30	6.7% (112/1,673)	10.9% (182/1,673)	1.9% (1/52)
	1.30-2.66	10.5% (68/648)	18.1% (117/648)	13.0% (3/23)
	≥2.67	27.1% (23/85)	35.3% (30/85)	33.3% (1/3)
Patients with DM (Calgary = 2,772) (Edmonton = 218)	<1.30	9.3% (160/1,730)	16.1% (278/1,730)	17.8% (24/135)
	1.30-2.66	21.0% (176/837)	31.9% (267/837)	47.1% (32/68)
	≥2.67	47.8% (98/205)	57.6% (118/205)	80.0% (12/15)
Patients without DM (Calgary = 5,354) (Edmonton = 767)	<1.30	3.9% (152/3,940)	8.3% (326/3,940)	6.0% (37/619)
	1.30-2.66	9.1% (109/1,205)	17.4% (210/1,205)	16.7% (22/132)
	≥2.67	28.2% (59/209)	38.3% (80/209)	43.8% (7/16)
Female patients (Calgary = 4,261) (Edmonton = 321)	<1.30	6.2% (191/3,070)	11.4% (349/3,070)	9.3% (20/216)
	1.30-2.66	12.6% (124/982)	20.7% (203/982)	34.1% (31/91)
	≥2.67	41.6% (87/209)	52.2% (109/209)	78.6% (11/14)
Male patients (Calgary = 3,852) (Edmonton = 664)	<1.30	4.6% (120/2,591)	9.8% (254/2,591)	7.6% (41/538)
	1.30-2.66	15.2% (160/1,056)	25.8% (272/1,056)	21.1% (23/109)
	≥2.67	34.2% (70/205)	43.4% (89/205)	47.1% (8/17)
Patients with BMI <35 (Calgary = 4,699) (Edmonton = 735)	<1.30	3.3% (108/3,253)	6.6% (216/3,253)	4.6% (26/569)
	1.30-2.66	9.2% (110/1,193)	17.6% (210/1,193)	18.9% (27/143)
	≥2.67	32.8% (83/253)	44.7% (113/253)	65.2% (15/23)
Patients with BMI ≥35 (Calgary = 1,845) (Edmonton = 250)	<1.30	10.9% (146/1,339)	19.7% (264/1,339)	18.9% (35/185)
	1.30-2.66	22.8% (98/430)	33.7% (145/430)	47.4% (27/57)
	≥2.67	47.4% (36/76)	52.6% (40/76)	50.0% (4/8)
Patient age ≥65 years (Calgary = 1,675) (Edmonton = 68)	<1.30	7.0% (41/582)	12.0% (70/582)	0% (0/8)
	1.30-2.66	13.8% (120/868)	23.2% (201/868)	29.6% (13/44)
	≥2.67	39.1% (88/225)	49.3% (111/225)	62.5% (10/16)
Patient age <65 years (Calgary = 6,451) (Edmonton = 917)	<1.30	5.3% (271/5,088)	10.5% (534/5,088)	8.2% (61/746)
	1.30-2.66	14.1% (165/1,174)	23.5% (276/1,174)	26.3% (41/156)
	≥2.67	36.5% (69/189)	46.0% (87/189)	60.0% (9/15)

2D-SWE, two-dimensional shear wave elastography; ALT, alanine aminotransferase; DM, diabetes mellitus type 2; FIB-4, fibrosis 4 variable score; VCTE, vibration-controlled transient elastography.

### Characteristics of the calgary MASLD cohort according to variables of interest

In the Calgary MASLD cohort, female patients were older, had higher BMI, and were more likely to have diabetes and comorbidities compared to males (Table S2). Patients with normal ALT were older, more likely female, and more likely to have ≥2 comorbidities compared to patients with elevated ALT (Table S3). Class 2/3 obesity (BMI ≥35 kg/m^2^) was prevalent in the Calgary MASLD pathway cohort (28%). Patients with BMI ≥35 were more likely to be younger, female, and have diabetes and comorbidities (Table S4). Patients with MASLD and diabetes were more likely to be older, female, and have higher BMI and more comorbidities (Table S5). Patients aged >65 years had lower BMI compared to patients aged <65 years and were more likely to have diabetes and comorbidities (Table S6). Laboratory data differences according to exposure variables are shown in the supplementary tables.

### Association between FIB-4 and 2D-SWE according to patient characteristics

**Fig. 2:**
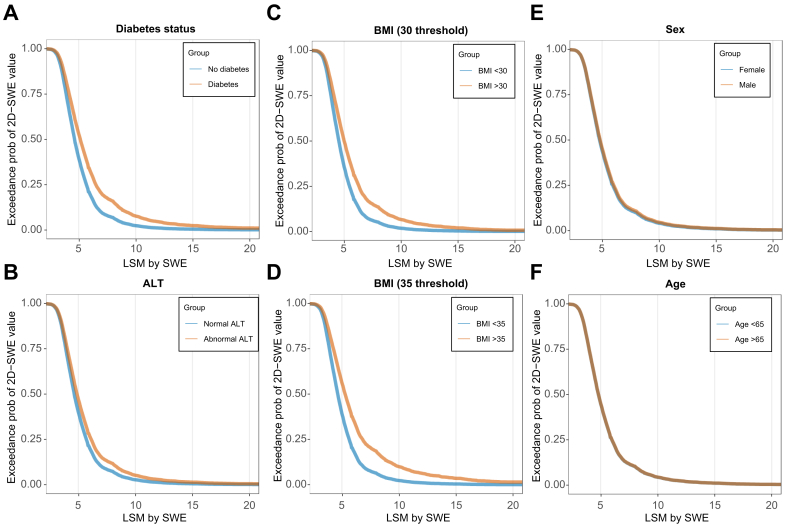
Exceedance probabilities of 2D-SWE for a FIB-4 value of 1.3. Each panel shows the exceedance probability, the probability that a patient’s 2D-SWE will be at or above the value on the x-axis (kPa). Curves compare two strata of the covariate named in each panel (A: diabetes; B: abnormal ALT; C: BMI with a 30 threshold; D: BMI with a 35 threshold; E: sex; F: age strata as shown in the panel legends). Higher curves indicate a greater chance of exceeding any given 2D-SWE threshold for the given 1.3 FIB-4 value. Estimates come from ordinal regression models (introducing one variable at a time) as explained in the methods and supplementary methods section. These findings suggest that a FIB-4 of 1.3 predicts higher values of 2D-SWE in individuals with diabetes, obesity and abnormal ALT, whereas the effect of age and sex is minor. 2D-SWE, shear-wave elastography; ALT, alanine aminotransferase; FIB-4, fibrosis-4 index.

Since the 2D-SWE threshold for referral to hepatology might evolve according to available treatments, guideline recommendations, and available resources, we modelled the association of FIB-4 values with the exceedance probabilities of any value of 2D-SWE, testing the influence of patient characteristics on FIB-4 predictions. As shown in Fig. 1, for a given FIB-4 value, patients with diabetes, BMI >35, and abnormal transaminases had a greater probability of having higher 2D-SWE values. On the other hand, age and sex did not have a major impact on FIB-4 predictions. As shown in Fig. 2, using a FIB-4 cut-off threshold of 1.30, there is risk of missing patients with 2D-SWE ≥8 kPa; however, the risk of missing high 2D-SWE values (*i.e*. >12 kPa) is very low, even in patients with unfavorable characteristics.

Furthermore, we developed a unified model to predict different 2D-SWE thresholds according to FIB-4 values and the five variables tested above. After backwards elimination, FIB-4, BMI, sex, diabetes and abnormal transaminases were retained in the model. Details of this model and instructions for calculating the exceedance probability of different 2D-SWE thresholds (with examples), together with a graphical representation of the model are presented in detail in the supplementary methods.

### Validation in the Edmonton MASLD cohort using VCTE

The Edmonton-MASLD cohort, with paired FIB-4/VCTE values in 985 patients, had a slightly different patient profile than the Calgary-MASLD cohort. Still, there was a similar impact of the clinical variables on FIB-4 predictions as we saw with the FIB4-2D-SWE cohort. Indeed, patients with diabetes, BMI >35, and abnormal transaminases showed a higher probability of having a VCTE ≥8 kPa at FIB-4 thresholds of 1.30 and 2.67 (Table 2). However, the probability of having a VCTE ≥8 kPa was substantially higher than the probability of having a 2D-SWE ≥8 kPa in the Calgary cohort and it was closer to 2D-SWE ≥6.5 kPa (Table 2).

**Fig. 3:**
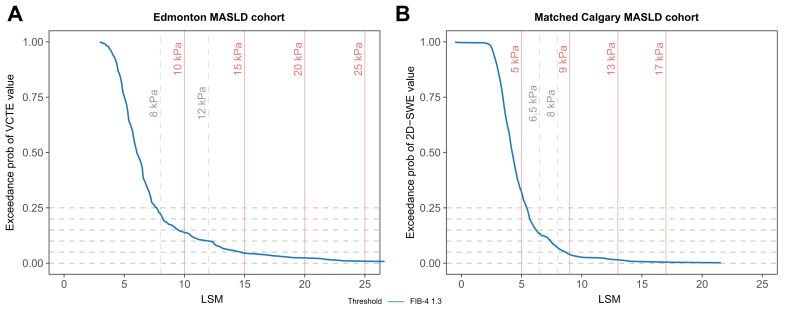
Exceedance probabilities of LSM for a FIB-4 value of 1.3. (A) VCTE in the Edmonton cohort. (B) 2D-SWE in the matched Calgary cohort. Horizontal dashed lines indicate reference probabilities (5%, 10%, 15%, 20%, and 25%). Vertical red lines represent the "rule of 5s" for VCTE, and the "rule of 4s" for 2D-SWE. Additional grey lines represent the 8 and 12 kPa VCTE threshold, and how these approximate 6.5 and 8 kPa thresholds for 2D-SWE in the matched Calgary sample. The figure illustrates that the same FIB-4 value of 1.3 predicts higher VCTE values than 2D-SWE values. For example, whereas a FIB-4 of 1.3 is associated with a 4% probability of finding a VCTE value ≥15 kPa in the Edmonton cohort, it is associated with a 4% probability of finding an 2D-SWE value ≥9 kPa in the Calgary cohort (see Results for details). As shown in the plot, the 9, 13, 17 values of the "rule of 4s" for SWE, might reflect the 15, 20 and 25 kPa values of the rule of 5s in VCTE. 2D-SWE, two-dimensional shear wave elastography; FIB-4, fibrosis-4 index; LSM, liver stiffness measurement; VCTE, vibration-controlled transient elastography.

To determine whether this difference was driven by distinct patient profiles between cohorts or by systematic differences between the two techniques, we modeled the association between FIB-4 and VCTE in the Edmonton cohort, and between FIB-4 and 2D-SWE in a subsample of the Calgary cohort matched 1:1 to the Edmonton cohort by age, sex, diabetes status, elevated transaminases, and BMI. Figure 3 presents the graphical representation of both models, including indicators for the VCTE “rule of 5s” thresholds (10, 15, 20, 25 kPa) and the World Federation for Ultrasound in Medicine and Biology (WFUMB) “rule of four” 2D-SWE thresholds (5, 9, 13, 17 kPa).^22^ The 15, 20, and 25 kPa VCTE thresholds corresponded approximately to the 9, 13, and 17 kPa 2D-SWE thresholds. Additionally, the 8 and 12 kPa VCTE thresholds corresponded approximately to 6.5 and 8 kPa 2D-SWE, respectively.

## Discussion

In this study, we describe the impact of clinical characteristics on current recommendations for risk-stratification of patients with MASLD. We evaluated two large cohorts identified from primary care-based MASLD pathways in Canada. To our knowledge, our study reports findings from two of the largest MASLD cohorts evaluated within primary care.^6^ Our cohort characteristics were different in terms of age, sex distribution and indications for MASLD risk stratification. However, they were comparable to other MASLD populations.[24], [25], [26] In our study, we addressed clinical questions on the impact of certain patient demographic and clinical characteristics that may affect performance of recommended MASLD clinical care pathways. In both of our cohorts, we observed that patients with obesity and diabetes have a much higher proportion of high elastography values for a given FIB-4 value.

A distinct aspect of our study is the inclusion of data from the Calgary MASLD pathway, which includes patients with metabolic risk factors beyond elevated transaminases. We reported risk stratification performance among patients with normal liver enzymes and class 2/3 obesity that were not available in previous clinical care pathways. Specifically, clinical care pathways that have been implemented worldwide to risk-stratify patients with MASLD in primary care have focused on evaluating patients who have either elevated ALT levels^27^^,^^28^ or metabolic dysfunction as a risk factor for MASLD.^14^^,^[29], [30], [31] These pathways used FIB-4, NAFLD fibrosis score, or the AST/ALT ratio as an initial step in patient risk stratification, followed by VCTE or ELF as a second test to confirm the presence of significant liver fibrosis. The observed prevalence of ≥8 kPa in our cohorts is aligned with risk-enriched clinical pathways, consistent with our cohort composition and referral strategy.^32^^,^^33^

Head-to-head comparisons of recent NITs showed cohort-specific performance. In Calgary, FIB-4 and LiverPRO had similar AUROC accuracy and exceeded FNI. However, in Edmonton, FNI outperformed FIB-4 and LiverPRO. We therefore retain FIB-4 as the default for simplicity and availability. International guidelines recommend using FIB-4 as an initial step for risk stratification. In our cohorts, ∼23-30% of patients with MASLD had a FIB-4 score ≥1.30, similar to previous findings in other pathways.^6^^,^^27^ As reported by Pierce *et al.*, there is significant variability between 2D-SWE machines, but each 2D-SWE system demonstrates reasonable reliability.^34^ Therefore, we used a single 2D-SWE machine in the Calgary cohort.

Our findings show how WFUMB ‘rule of four’ thresholds map onto commonly used VCTE cut-offs in MASLD referral pathways. In our data, 2D-SWE values of 9–13–17 kPa aligned in an expected stepwise fashion with VCTE thresholds of 15-20 and 25 kPa, supporting the use of established WFUMB thresholds to harmonize elastography-based triage across modalities. To validate these findings, we matched both cohorts (Calgary and Edmonton 1:1 by age, sex, diabetes, abnormal transaminases and BMI) and preformed an indirect comparison of 2D-SWE and VCTE by anchoring both values on FIB-4, assuming that in the matched cohorts, FIB-4 predictions should be roughly equivalent. In the matched Calgary cohort, a FIB-4 of 1.30 yielded a 10% exceedance probability of having a 2D-SWE ≥8 kPa, whereas in the Edmonton cohort, it gave a 10% exceedance probability of having a VCTE ≥12 kPa. This indirect comparison suggests that a 2D-SWE threshold of 8 kPa could be roughly equivalent to a VCTE threshold of 12 kPa. These findings confirm previous reports that clinically relevant thresholds for 2D-SWE are lower than with VCTE, and refinement of decision thresholds is essential in implementing 2D-SWE-based risk-stratification on a large scale.^11^^,^^22^

In our study, we evaluated the influence of sex and age on the performance of FIB-4 followed by LSM measured using either 2D-SWE or VCTE. Although MASLD is more prevalent among males, females more commonly have advanced fibrosis, particularly after menopause.^35^ Our cohorts differed in sex distribution; however, LSM values measured by both 2D-SWE and VCTE were higher in females across all FIB-4 categories. Interestingly, after adjusting for BMI, diabetes and abnormal ALT, sex had only a minor predictive value and, in fact, male sex predicted higher 2D-SWE values for a given FIB-4. These findings emphasize the importance of examining sex-specific differences in non-invasive fibrosis testing and the need for sex-oriented referral pathways for MASLD.

Age is a known predictor of fibrosis progression in MASLD and other chronic liver diseases. Because older age increases FIB-4 values, current guidelines recommend using higher FIB-4 cut-offs for patients older than 65 years. Elevated LSM measured by either 2D-SWE or VCTE was similar between patients stratified by the 65-year age threshold. Furthermore, in our FIB-4 based regression models, age did not predict elevated 2D-SWE or VCTE values, consistent with our previous findings.^15^

Most patients in our MASLD cohorts had elevated ALT at baseline. However, one third of patients assessed in the Calgary MASLD cohort had normal ALT. Interestingly, patients with normal ALT had higher BMI and lower prevalence of diabetes, compared to those with elevated ALT. LSMs were higher across all categories of FIB-4 among patients with elevated ALT compared to patients with normal ALT. Therefore, in our predicting elevated LSM models, elevated transaminases were a significant predictor.

Obesity was prevalent in our cohorts, with a median BMI of 31.2 and 30.9 kg/m^2^ in the Calgary and Edmonton cohorts, respectively. This distinguishes our study from many published cohorts from Europe and Asia who included patient populations with lower BMIs.^6^ We show that BMI has a significant impact on the association between FIB-4 and 2D-SWE or VCTE values, questioning the use of the same FIB-4 threshold (1.30) for initial referral triage in patients with class 2/3 obesity.^36^ A portion of the higher exceedance we observed in obesity class 2/3 may reflect measurement bias rather than true histologic fibrosis. Obesity increases skin-to-liver distance and alters shear-wave propagation, which can inflate elastography values.^37^ Consistent with this, some liver biopsy-based cohorts have reported inverse or no relationships between BMI and fibrosis stage.^38^^,^^39^ While obesity class 2/3 is associated with higher rates of significant and advanced fibrosis measured by LSM in our cohorts, it is recommended to repeat elastography or use further tests such as magnetic resonance elastography or liver biopsy to confirm fibrosis stage among these patients.^40^^,^^41^

The recognition that diabetes is a major risk factor for developing advanced fibrosis among patients with MASLD is growing.^42^^,^^43^ Similar to our study, a recent meta-analysis identified that patients with diabetes have two-times the risk of developing advanced liver fibrosis.^42^ In our cohort, the proportion of patients with a FIB-4 <1.30 who had high 2D-SWE values was much higher in patients with diabetes than without diabetes. Our findings support recent recommendations calling for a fast-track or dedicated pathway to assess liver fibrosis in patients with MASLD and diabetes, who have the highest prevalence of advanced fibrosis.^44^^,^^45^

Our study has some limitations. We addressed potential selection bias from AST ordering and FIB-4 availability by explicitly comparing patients with *vs.* without FIB-4; groups were broadly similar, with near-identical median 2D-SWE. In our jurisdiction, patients with suspected alcohol-related liver disease (ALD) are routinely triaged through a dedicated ALD pathway, which likely diverts patients with ALD away from the MASLD pathway. Consequently, our primary-care cohort reflects a blended MASLD/MetALD population. We recognize that using 2D-SWE may not be feasible in other jurisdictions with limited resources, or with ultrasound providers using different types of 2D-SWE machines. To mitigate this limitation, we collaborated with a radiology group well trained in 2D-SWE and who used a single type of 2D-SWE machine. Furthermore, we used an external cohort with paired FIB-4 and VCTE that allowed us to indirectly benchmark the interpretation of 2D-SWE in relation to VCTE values. Validation of our findings using different 2D-SWE machines are warranted.^46^

In conclusion, in two large North American MASLD cohorts assessed in primary care, we show that initial triage with FIB-4 requires refinement for individuals with obesity and diabetes, because a substantial proportion of patients with elevated liver stiffness may be missed. This issue is particularly relevant for selecting MASLD patients for newly available pharmacologic treatments, which are largely based on NITs indicating advanced fibrosis.

## Abbreviations

2D-SWE, two-dimensional shear wave elastography; ALD, alcohol-related liver disease; ALT, alanine aminotransferase; AST, aspartate aminotransferase; FIB-4, fibrosis-4 index; FNI, fibrotic NASH index; HbA1c, hemoglobin A1c; INR, international normalized ratio; LSM, liver stiffness measurement; MASLD, metabolic dysfunction-associated steatotic liver disease; MetALD, metabolic dysfunction- and alcohol-associated liver disease; VCTE, vibration-controlled transient elastography.

## Authors’ contributions

AAS, EB and JGA designed the study. AAS, EB, MK, MT, MMM, JJ, FL, WS, AM and JGA collected and analyzed the data. AAS, EB, MS, GS and JGA drafted the manuscript. All Authors interpreted the data and provided critical revisions of the manuscript for important intellectual contents. All authors have approved the final draft of the manuscript.

## Data availability

The data supporting the findings of this study are not publicly available because of ethics and privacy restrictions. De-identified data may be shared upon reasonable request and with approval from the University of Calgary Research Ethics Board (ethics committee), and subject to any required institutional data access agreements.

## Financial support

This work was funded through a Canadian Institutes of Health Research (10.13039/501100000024CIHR) Project Grant and a Gilead Sciences investigator-initiated grant awarded to Dr. A Shaheen.

## Conflicts of interest

AAS has participated in advisory boards and received research grants from Gilead, Novo Nordisk and Intercept. JGA received grants from Cook and Gilead (paid to the University of Alberta) and received consulting fees from Boehringer Ingelheim, AstraZeneca, Inventiva and 89Bio.

Please refer to the accompanying ICMJE disclosure forms for further details.

## References

[1] Riazi K., Azhari H., Charette J.H. (2022). The prevalence and incidence of NAFLD worldwide: a systematic review and meta-analysis. Lancet Gastroenterol Hepatol.

[2] Rinella M.E., Lazarus J.V., Ratziu V. (2023). A multi-society Delphi consensus statement on new fatty liver disease nomenclature. Ann Hepatol.

[3] Hagstrom H., Nasr P., Ekstedt M. (2017). Fibrosis stage but not NASH predicts mortality and time to development of severe liver disease in biopsy-proven NAFLD. J Hepatol.

[4] Mantovani A., Csermely A., Petracca G. (2021). Non-alcoholic fatty liver disease and risk of fatal and non-fatal cardiovascular events: an updated systematic review and meta-analysis. Lancet Gastroenterol Hepatol.

[5] Kanwal F., Shubrook J.H., Adams L.A. (2021). Clinical care pathway for the risk stratification and management of patients with nonalcoholic fatty liver disease. Gastroenterology.

[6] Abeysekera K.W.M., Macpherson I., Glyn-Owen K. (2022). Community pathways for the early detection and risk stratification of chronic liver disease: a narrative systematic review. Lancet Gastroenterol Hepatol.

[7] Lazarus J.V., Anstee Q.M., Hagstrom H. (2021). Defining comprehensive models of care for NAFLD. Nat Rev Gastroenterol Hepatol.

[8] Tavaglione F., Jamialahmadi O., De Vincentis A. (2023). Development and validation of a score for fibrotic nonalcoholic steatohepatitis. Clin Gastroenterol Hepatol.

[9] Noureddin M., Charlton M.R., Harrison S.A. (2024). Expert panel recommendations: practical clinical applications for initiating and monitoring resmetirom in patients with MASH/NASH and moderate to noncirrhotic advanced fibrosis. Clin Gastroenterol Hepatol.

[10] Chen V.L., Morgan T.R., Rotman Y. (2025). Resmetirom therapy for metabolic dysfunction-associated steatotic liver disease: october 2024 updates to AASLD Practice Guidance. Hepatology.

[11] European Association for the Study of the Liver, European Association for the Study of D, European Association for the Study of O (2024). EASL-EASD-EASO Clinical Practice Guidelines on the management of metabolic dysfunction-associated steatotic liver disease (MASLD): executive Summary. Diabetologia.

[12] Kim R.G., Deng J., Reaso J.N. (2022). Noninvasive fibrosis screening in fatty liver disease among vulnerable populations: impact of diabetes and obesity on FIB-4 score accuracy. Diabetes Care.

[13] Davyduke T., Tandon P., Al-Karaghouli M. (2019). Impact of implementing a "FIB-4 first" strategy on a pathway for patients with NAFLD referred from primary care. Hepatol Commun.

[14] Shaheen A.A., Riazi K., Medellin A. (2020). Risk stratification of patients with nonalcoholic fatty liver disease using a case identification pathway in primary care: a cross-sectional study. CMAJ Open.

[15] Sung S., Al-Karaghouli M., Tam M. (2025). Age-dependent differences in FIB-4 predictions of fibrosis in patients with MASLD referred from primary care. Hepatol Commun.

[16] Kim D.W., Suh C.H., Kim K.W. (2019). Technical performance of two-dimensional shear wave elastography for measuring liver stiffness: a systematic review and meta-analysis. Korean J Radiol.

[17] Kim H.C., Nam C.M., Jee S.H. (2004). Normal serum aminotransferase concentration and risk of mortality from liver diseases: prospective cohort study. BMJ.

[18] Lindvig K.P., Thorhauge K.H., Hansen J.K. (2025). Development, validation, and prognostic evaluation of LiverPRO for the prediction of significant liver fibrosis in primary care: a prospective cohort study. Lancet Gastroenterol Hepatol.

[19] DeLong E.R., DeLong D.M., Clarke-Pearson D.L. (1988). Comparing the areas under two or more correlated receiver operating characteristic curves: a nonparametric approach. Biometrics.

[20] Quan H., Li B., Couris C.M. (2011). Updating and validating the Charlson comorbidity index and score for risk adjustment in hospital discharge abstracts using data from 6 countries. Am J Epidemiol.

[21] Liu Q., Shepherd B.E., Li C. (2017). Modeling continuous response variables using ordinal regression. Stat Med.

[22] Barr R.G., Wilson S.R., Rubens D. (2020). Update to the society of radiologists in ultrasound liver elastography consensus statement. Radiology.

[23] Ho D., Imai K., King G. (2011). MatchIt: nonparametric preprocessing for parametric causal inference. J Stat Softw.

[24] Kim D., Konyn P., Sandhu K.K. (2021). Metabolic dysfunction-associated fatty liver disease is associated with increased all-cause mortality in the United States. J Hepatol.

[25] Zhang X., Heredia N.I., Balakrishnan M. (2021). Prevalence and factors associated with NAFLD detected by vibration controlled transient elastography among US adults: results from NHANES 2017-2018. PLoS One.

[26] Yip T.C., Lee H.W., Lin H. (2025). Prognostic performance of the two-step clinical care pathway in metabolic dysfunction-associated steatotic liver disease. J Hepatol.

[27] Srivastava A., Gailer R., Tanwar S. (2019). Prospective evaluation of a primary care referral pathway for patients with non-alcoholic fatty liver disease. J Hepatol.

[28] Macpherson I., Nobes J.H., Dow E. (2020). Intelligent liver function testing: working smarter to improve patient outcomes in liver disease. J Appl Lab Med.

[29] El-Gohary M., Moore M., Roderick P. (2018). Local care and treatment of liver disease (LOCATE) - a cluster-randomized feasibility study to discover, assess and manage early liver disease in primary care. PLoS One.

[30] Mansour D., Grapes A., Herscovitz M. (2021). Embedding assessment of liver fibrosis into routine diabetic review in primary care. JHEP Rep.

[31] Hayward K.L., McKillen B.J., Horsfall L.U. (2022). Towards collaborative management of non-alcoholic fatty liver disease: a 'real-world' pathway for fibrosis risk assessment in primary care. Intern Med J.

[32] Gines P., Castera L., Lammert F. (2022). Population screening for liver fibrosis: toward early diagnosis and intervention for chronic liver diseases. Hepatology.

[33] Harris R., Card T.R., Delahooke T. (2019). Obesity is the most common risk factor for chronic liver disease: results from a risk stratification pathway using transient elastography. Am J Gastroenterol.

[34] Pierce T.T., Ozturk A., Sherlock S.P. (2024). Reproducibility and repeatability of US shear-wave and transient elastography in nonalcoholic fatty liver disease. Radiology.

[35] Balakrishnan M., Patel P., Dunn-Valadez S. (2021). Women have a lower risk of nonalcoholic fatty liver disease but a higher risk of progression vs men: a systematic review and meta-analysis. Clin Gastroenterol Hepatol.

[36] Qadri S., Ahlholm N., Lonsmann I. (2022). Obesity modifies the performance of fibrosis biomarkers in nonalcoholic fatty liver disease. J Clin Endocrinol Metab.

[37] European Association for the Study of the Liver (2021). EASL Clinical Practice Guidelines on non-invasive tests for evaluation of liver disease severity and prognosis - 2021 update. J Hepatol.

[38] Hirose S., Matsumoto K., Tatemichi M. (2020). Nineteen-year prognosis in Japanese patients with biopsy-proven nonalcoholic fatty liver disease: lean versus overweight patients. PLoS One.

[39] Drolz A., Wolter S., Wehmeyer M.H. (2021). Performance of non-invasive fibrosis scores in non-alcoholic fatty liver disease with and without morbid obesity. Int J Obes (Lond).

[40] Quek J., Chan K.E., Wong Z.Y. (2023). Global prevalence of non-alcoholic fatty liver disease and non-alcoholic steatohepatitis in the overweight and obese population: a systematic review and meta-analysis. Lancet Gastroenterol Hepatol.

[41] Chouik Y., Aubin A., Maynard-Muet M. (2024). The grade of obesity affects the noninvasive diagnosis of advanced fibrosis in individuals with MASLD. Obesity (Silver Spring).

[42] Jarvis H., Craig D., Barker R. (2020). Metabolic risk factors and incident advanced liver disease in non-alcoholic fatty liver disease (NAFLD): a systematic review and meta-analysis of population-based observational studies. Plos Med.

[43] Park H., Cheuk-Fung Yip T., Yoon E.L. (2025). Impact of cardiometabolic risk factors on hepatic fibrosis and clinical outcomes in MASLD: a population-based multi-cohort study. JHEP Rep.

[44] Blank V., Petroff D., Beer S. (2020). Current NAFLD guidelines for risk stratification in diabetic patients have poor diagnostic discrimination. Sci Rep.

[45] Udompap P., Therneau T.M., Canning R.E. (2023). Performance of American Gastroenterological Association Clinical Care Pathway for the risk stratification of patients with nonalcoholic fatty liver disease in the US population. Hepatology.

[46] Congly S.E., Shaheen A.A., Swain M.G. (2021). Modelling the cost effectiveness of non-alcoholic fatty liver disease risk stratification strategies in the community setting. PLoS One.

