# Supplementary material for: Diabetes and obesity reduce FIB-4 accuracy in MASLD referral pathways
Source: JHEP Rep. 2026 Jan 12;8(4):101735. doi: 10.1016/j.jhepr.2026.101735 (PMC12969413; doi:10.1016/j.jhepr.2026.101735)
Supplement: Multimedia component 1 [file mmc1.pdf]

Diabetes and obesity reduce FIB-4 accuracy in MASLD referral pathways

Abdel-Aziz Shaheen, Elizabeth Baguley, Mark G Swain, Matthew Tam, Mang Ming Ma, Giada Sebastiani, Jason Jiang, Frank Lee, Alexandra Medellin, Juan G Abraldes

Table of contents

Model building process……………………………………………………………………2

Results of the matching process…………………………………………………………4

Fig. S1………………………………………………………………………………………5

Table S1……………………………………………………………………………………6

Table S2……………………………………………………………………………………7

Table S3……………………………………………………………………………………8

Table S4……………………………………………………………………………………9

Table S5…………………………………………………………………………………...10

Table S6…………………………………………………………………………………...11

Table S7…………………………………………………………………………………...12

Model building process

The modeling process, based on ordinal regression for a continuous outcome, was conducted following methods and code described in https://hbiostat.org/rmsc/cony#ordinal-regression-models-for-continuous-y.

The goal of the modeling process was to assess how the variables of interest contribute to the prediction of SWE, which is primarily a descriptive modeling question. The log-log link was chosen since it provided a better fit to the data than the logit link. FIB-4 was modeled with restricted cubic splines (4 knots). To show the effects of these variables on SWE predictions we plotted the exceedance probabilities of different values of SWE for the commonly used FIB-4 threshold values of 1.3 and 2.67.

To develop a unified model to predict different SWE thresholds according to FIB-4 and the 5 variables of interest we used a similar approach. After backwards elimination (with AIC as stopping rule, i.e., (i.e., a factor is deleted if the $\chi 2$ falls below twice its degrees of freedom) FIB-4, BMI (both modeled with restricted cubic splines with 4 knots), sex, diabetes and abnormal transaminases were retained in the model.

The table shows the relative explained variation (with 95% CI) of each variable to explain the variability in SWE

Relative Explained Variation (REV)

	REV	Lower CI	Upper CI
FIB4	0.435	0.377	0.504
Sex	0.013	0.005	0.028
BMI	0.319	0.282	0.375
diabetes	0.118	0.071	0.145
abnormal ALT	0.029	0.011	0.046

The nomogram below provides a graphical representation of the model. The nomogram translates the multivariable ordinal regression model into a point-based tool that allows to grasp the relative contribution of each variable to the model. To use the nomogram: (1) For each predictor (FIB-4, BMI, sex, diabetes, abnormal ALT), locate the patient’s value on its axis. (2) Draw a vertical line to the “Points” axis to assign points for that predictor. (3) Sum points across all predictors to obtain the “Total Points.” (4) Locate the “Total Points” on the corresponding axis and draw a vertical line downward to read the exceedance probabilities for each threshold of SWE (7,8,10,13 and 17 kPa are shown as examples) on the bottom scales. For example, a patient with a FIB4 of 1.3 (˜41 points), Male (˜10 points), with a BMI of 35 (˜34 points), with diabetes (˜33 points) and abnormal ALT (˜15 points), would have a total of ˜133 points. That would give the patient a 25% chances *(exceedance probability*) of having a SWE of 7 kPa or more , a 19% of 8 kPa or more , and 11% of 10 kPa or more, a 5% of 13 kPa or more and a 2% of having 17 kPa or more.

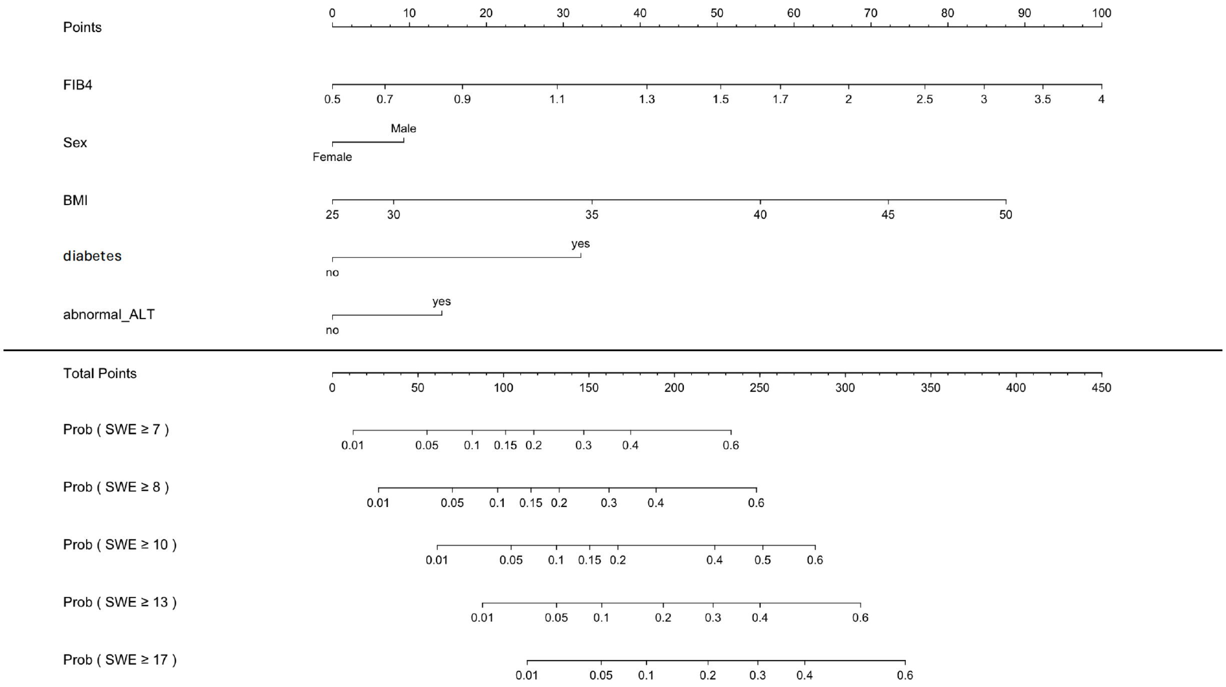

The formula to calculate the exact exceedance probabilities for a given SWE threshold is as follows:

Exceedance probability of having (SWE>=Y) = exp(-exp(-linear_predictor))

Where the linear_predictor is calculated as follows

Linear_predictor = Intercept (specific for every SWE value) +

0.2603354 * FIB4 +

0.3864207 * max(FIB4 - 0.38199998, 0)^3 -

0.94166768 * max(FIB4 - 0.78029412, 0)^3 +

0.61079383 * max(FIB4 - 1.2038862, 0)^3 -

0.055546848 * max(FIB4 - 2.6673195, 0)^3 +

0.099700558 * (Sex == "Male") +

0.0068417906 * BMI +

0.00027493356 * max(BMI - 23.23, 0)^3 -

0.00074095161 * max(BMI - 29.04, 0)^3 +

0.00051799554 * max(BMI - 33.66, 0)^3 -

5.1977492e-05 * max(BMI - 44.35, 0)^3 +

0.31463483 * (diabetes == "yes") +

0.16062209 * (abnormal ALT == "yes")

Intercepts for selected thresholds of SWE

y>=6.5	-1.8059
y>=7	-1.9485
y>=8	-2.0948
y>=9	-2.2944
y>=10	-2.4183
y>=11	-2.5179
y>=12	-2.5985
y>=13	-2.6786
y>=14	-2.7401
y>=15	-2.7940
y>=16	-2.8537
y>=17	-2.9323

Results of the matching process

Matching between Edmonton and Calgary cohorts were performed as described in methods in the main manuscript.
The different characteristics of the two cohorts are shown in table 1
The following table show the descriptives of the sample after matching

Post Matching

	Means Edmonton	Means Calgary	Std. Mean Diff.
diabetes	0.2213	0.2122	0.0220
BMI	31.5960	31.6129	-0.0030
abnormal_ALT	0.9147	0.9025	0.0436
age	44.1878	43.9513	0.0185
FemaleSex	0.3259	0.3239	0.0043

Fig. S1: Inclusion and exclusion criteria for the Calgary and Edmonton MASLD cohorts

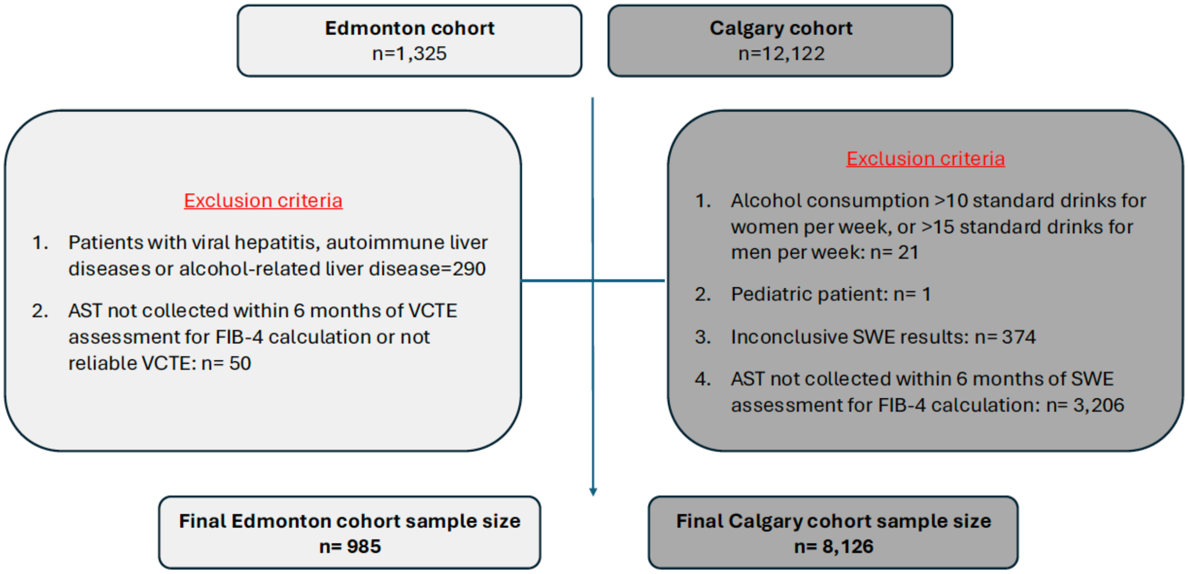

Table S1: Comparison of patient characteristics of the Calgary MASLD pathway according to FIB-4 availability

Characteristic	Calgary MASLD cohort patients with FIB-4 N=8,126	Calgary MASLD cohort patients without FIB-4 N=3,532	P value
Age, yrs.	54 (43-63)	55 (44-63)	0.125
Female sex	52.5% (4,261)	49.2% (1,732)	0.001
BMI (Kg/Height in meter^2)	31.2 (27.6-35.7)	31.5 (27.9-35.9)	0.070
Baseline investigations			
Albumin, g/L	39 (37-41)	39 (37-41)	0.043
Alkaline Phosphatase, U/L	77 (64-95)	76 (63-93)	<0.001
Triglycerides, mmol/L	1.8 (1.2-2.5)	1.7 (1.3-2.5)	0.522
HDL, mmol/L	1.2 (1.0-1.4)	1.2 (1.0-1.4)	0.151
LDL, mmol/L	2.6 (1.9-3.3)	2.7 (2.0-3.3)	0.002
Total bilirubin, μmol/L	8 (6-11)	8 (6-11)	0.089
HbA1c, %	5.8 (5.5-6.2)	5.8 (5.5-6.2)	0.086
Diabetes mellitus	34.1% (2,772)	31.9% (1,128)	0.022
Comorbidities, Charlson Index			<0.001
0	34.4% (2,791)	39.6% (1,398)	
1	21.8% (1,769)	21.8% (771)	
≥ 2	43.9% (3,566)	38.6% (1,363)	
SWE, valid measurements in kPa	4.6 (3.8-5.7)	4.5 (3.8-5.4)	<0.001

Data presented as median (IQR) or % (n). BMI, body mass index; ALT, alanine aminotransferase; AST, aspartate aminotransferase; HDL, high density lipoprotein; LDL, low density lipoprotein; HbA1C, Hemoglobin A1C; SWE, shearwave elastography; FIB-4, fibrosis 4 variable score.

Table S2: Characteristics of the Calgary MASLD clinic patients according to sex.

Characteristic	Female patients<br>N=4,261, 52.5%	Male patients<br>N=3,852, 47.5%	P value
Age, yrs.	55 (46-63)	52 (41-62)	<0.001
BMI (Kg/Height in meter^2)	31.6 (27.4-36.7)	30.9 (27.8-34.7)	<0.001
Baseline investigations			
ALT, U/L	34 (22-53)	43 (28-68)	<0.001
AST, U/L	27 (20-38)	29 (22-41)	<0.001
Albumin, g/L	38 (36-41)	40 (38-42)	<0.001
Alkaline Phosphatase, U/L	82 (68-101)	73 (61-88)	<0.001
Platelets, 10E^9/L	269 (229-315)	232 (196-273)	<0.001
Triglycerides, mmol/L	1.7 (1.2-2.4)	1.8 (1.3-2.7)	<0.001
HDL, mmol/L	1.3 (1.1-1.5)	1.1 (0.9-1.2)	<0.001
LDL, mmol/L	2.7 (2.0-3.3)	2.5 (1.8-3.2)	<0.001
Total bilirubin, μmol/L	7 (5-10)	10 (7-13)	<0.001
HbA1C, %	5.8 (5.5-6.2)	5.7 (5.5-6.2)	<0.001
Diabetes mellitus	35.6% (1,516)	32.5% (1,251)	0.003
Comorbidities, Charlson Index			
0	30.2% (1,286)	38.9% (1,499)	<0.001
1	22.5% (958)	21.0% (810)	
≥ 2	47.3% (2,017)	40.1% (1,543)	
SWE, valid measurements in kPa	4.5 (3.8-5.7)	4.7 (3.9-5.8)	<0.001
SWE ≥ 8 kPa	9.4% (402)	9.1% (350)	0.589
FIB-4	0.95 (0.66-1.37)	0.99 (0.68-1.47)	<0.001
FIB-4 ≥ 1.30	28.0% (1,191)	32.7% (1,261)	<0.001
FIB-4 ≥ 2.65	4.9% (209)	5.3% (205)	0.394

Data presented as median (IQR) or % (n). BMI, body mass index; ALT, alanine aminotransferase; AST, aspartate aminotransferase; HDL, high density lipoprotein; LDL, low density lipoprotein; HbA1C, Hemoglobin A1C; SWE, shearwave elastography; FIB-4, fibrosis 4 variable score.

Table S3: Patients characteristics of the Calgary MASLD clinic according to normality of ALT at baseline.

Characteristic	Patients with normal ALT<br><br>N=2,406, 29.6%	Patients with elevated ALT<br><br>N=5,720, 70.4%	P value
Age, yrs.	57 (46-66)	53 (42-61)	<0.001
Female sex	55.8% (1,335)	51.2% (2,926)	<0.001
BMI (Kg/Height in meter^2)	30.9 (26.9-35.6)	31.4 (27.8-35.7)	0.008
Baseline investigations			
ALT, U/L	20 (16-23)	50 (36-71)	<0.001
AST, U/L	20 (17-24)	33 (25-46)	<0.001
Albumin, g/L	38 (36-41)	40 (37-42)	<0.001
Alkaline Phosphatase, U/L	74 (62-91)	79 (65-97)	<0.001
Platelets, 10E^9/L	256 (212-301)	249 (209-295)	<0.001
Triglycerides, mmol/L	1.6 (1.1-2.3)	1.8 (1.3-2.6)	<0.001
HDL, mmol/L	1.2 (1.0-1.4)	1.1 (1.0-1.4)	<0.001
LDL, mmol/L	2.5 (1.9-3.2)	2.6 (2.0-3.3)	<0.001
Total bilirubin, µmol/L	8 (5-11)	8 (6-12)	<0.001
HbA1C, %	5.7 (5.4-6.1)	5.8 (5.5-6.2)	<0.001
Diabetes mellitus	33.2% (798)	34.5% (1,974)	0.244
Comorbidities, Charlson Index<br>0<br>1<br>≥ 2	 31.1% (749)<br>23.1% (556)<br>45.8% (1,101)	 35.7% (2,042)<br>21.2% (1,213)<br>43.1% (2,465)	<0.001
SWE, valid measurements in kPa<br>SWE ≥ 8 kPa	4.4 (3.7-5.5)<br>8.4% (203)	4.7 (3.9-5.8)<br>9.6% (551)	<0.001<br>0.090
FIB-4<br>FIB-4 ≥ 1.30<br>FIB-4 ≥ 2.67	1.00 (0.70-1.41)<br>30.5% (733)<br>3.5% (85)	0.95 (0.66-1.42)<br>30.1% (1,723)<br>5.8% (329)	0.007<br>0.758<br><0.001

Data presented as median (IQR) or % (n). BMI, body mass index; ALT, alanine aminotransferase; AST, aspartate aminotransferase; HDL, high density lipoprotein; LDL, low density lipoprotein; HbA1C, Hemoglobin A1C; SWE, shearwave elastography; FIB-4, fibrosis 4 variable score.

Table S4: Characteristics of the Calgary MASLD clinic patients according to body mass index (BMI) cutoff 35.

Characteristic	Patients with BMI <35<br><br>N=4,699, 71.8%	Patients with BMI ≥35<br>N=1,845, 28.2%	P value
Age, yrs.	55 (44-64)	52 (42-61)	<0.001
Female sex	50.1% (2,351)	61.0% (1,124)	<0.001
Baseline investigations			
ALT, U/L	38 (25-60)	38 (25-60)	0.733
AST, U/L	28 (21-39)	27 (20-40)	0.155
Albumin, g/L	40 (38-42)	38 (36-41)	<0.001
Alkaline Phosphatase, U/L	76 (63-94)	79 (65-96)	<0.001
Platelets, $10E^9$/L	250 (209-295)	254 (215-305)	0.002
Triglycerides, mmol/L	1.8 (1.2-2.5)	1.8 (1.3-2.5)	0.001
HDL, mmol/L	1.2 (1.0-1.4)	1.1 (0.9-1.3)	<0.001
LDL, mmol/L	2.7 (2.0-3.3)	2.5 (1.9-3.2)	<0.001
Total bilirubin, μmol/L	8 (6-12)	8 (6-11)	<0.001
HbA1C, %	5.7 (5.5-6.1)	5.9 (5.5-6.4)	<0.001
Diabetes mellitus	30.3% (1,423)	42.2% (778)	<0.001
Comorbidities, Charlson Index<br>0<br>1<br>≥ 2	 36.9% (1,733)<br>22.4% (1,053)<br>40.7% (1,913)	 28.0% (517)<br>19.5% (359)<br>52.5% (969)	<0.001
SWE, valid measurements in kPa<br>SWE ≥ 8 kPa	4.4 (3.7-5.4)<br>6.4% (301)	5.1 (4.2-6.4)<br>15.2% (280)	<0.001<br><0.001
FIB-4<br>FIB-4 ≥ 1.30<br>FIB-4 ≥ 2.67	0.98 (0.69-1.43)<br>30.8% (1,446)<br>5.4% (253)	0.92 (0.63-1.36)<br>27.4% (506)<br>4.1% (76)	<0.001<br>0.008<br>0.035

Data presented as median (IQR) or % (n). BMI, body mass index; ALT, alanine aminotransferase; AST, aspartate aminotransferase; HDL, high density lipoprotein; LDL, low density lipoprotein; HbA1C, Hemoglobin A1C; SWE, shearwave elastography; FIB-4, fibrosis 4 variable score.

Table S5: Characteristics of the Calgary MASLD clinic patients according to body mass index (BMI) cutoff 30.

Characteristic	Patients with BMI <30<br>N=2,718, 41.6%	Patients with BMI ≥30<br>N=3,824, 58.5%	P value
Age, yrs.	55 (45-64)	54 (43-62)	<0.001
Female sex	51.4% (1,393)	54.5% (2,080)	0.012
Baseline investigations			
ALT, U/L	37 (24-58)	39 (25-61)	0.003
AST, U/L	28 (21-38)	28 (21-40)	0.955
Albumin, g/L	40 (38-42)	39 (37-41)	<0.001
Alkaline Phosphatase, U/L	76 (63-93)	78 (65-95)	0.001
Platelets, $10E^9/L$	249 (209-296)	253 (211-299)	0.022
Triglycerides, mmol/L	1.7 (1.2-2.4)	1.8 (1.3-2.6)	<0.001
HDL, mmol/L	1.2 (1.0-1.5)	1.1 (0.9-1.3)	<0.001
LDL, mmol/L	2.7 (2.0-3.3)	2.6 (1.9-3.2)	<0.001
Total bilirubin, μmol/L	9 (6-12)	8 (6-11)	<0.001
HbA1C, %	5.7 (5.4-6.1)	5.8 (5.5-6.3)	<0.001
Diabetes mellitus	28.6% (777)	37.2% (1,423)	<0.001
Comorbidities, Charlson Index			<0.001
0	38.6% (1,050)	31.4% (1,199)	
1	22.4% (608)	21.0% (804)	
≥ 2	39.0% (1,060)	47.6% (1,821)	
SWE, valid measurements in kPa	4.3 (3.7-5.2)	4.8 (4.0-6.0)	<0.001
SWE ≥ 8 kPa	5.5% (150)	11.3% (431)	<0.001
FIB-4	1.00 (0.69-1.45)	0.94 (0.65-1.38)	<0.001
FIB-4 ≥ 1.30	31.4% (853)	28.7% (1,099)	0.021
FIB-4 ≥ 2.67	5.6% (152)	4.6% (177)	0.079

Data presented as median (IQR) or % (n). BMI, body mass index; ALT, alanine aminotransferase; AST, aspartate aminotransferase; HDL, high density lipoprotein; LDL, low density lipoprotein; HbA1C, Hemoglobin A1C; SWE, shearwave elastography; FIB-4, fibrosis 4 variable score.

Table S6: Characteristics of the Calgary MASLD clinic patients according to having diabetes mellitus.

Characteristic	Patients without diabetes<br><br>N=5,354 , 65.9%	Patients with diabetes<br><br>N=2,772, 34.1%	P value
Age, yrs.	52 (41-61)	58 (48-66)	<0.001
Female sex	51.4% (2,754)	54.8% (1,516)	0.003
BMI (Kg/Height in meter^2)	30.7 (27.3-34.8)	32.3 (28.3-37.5)	<0.001
Baseline investigations			
ALT, U/L	38 (24-61)	39 (25-60)	0.387
AST, U/L	28 (21-39)	28 (21-41)	0.101
Albumin, g/L	39 (37-42)	39 (37-41)	<0.001
Alkaline Phosphatase, U/L	77 (64-95)	78 (65-96)	0.015
Platelets, 10E^9/L	254 (214-297)	248 (203-297)	<0.001
Triglycerides, mmol/L	1.7 (1.2-2.4)	1.9 (1.3-2.6)	<0.001
HDL, mmol/L	1.2 (1.0-1.4)	1.1 (0.9-1.3)	<0.001
LDL, mmol/L	2.8 (2.2-3.4)	2.2 (1.5-2.9)	<0.001
Total bilirubin, μmol/L	8 (6-12)	8 (6-11)	0.217
HbA1C, %	5.6 (5.4-5.8)	6.6 (6.1-7.4)	<0.001
Comorbidities, Charlson Index<br>0<br>1<br>≥ 2	 50.0% (2,675)<br>31.6% (1,694)<br>18.4% (985)	 4.2% (116)<br>2.7% (75)<br>93.1% (2,581)	<0.001
SWE, valid measurements in kPa<br>SWE ≥ 8 kPa	4.4 (3.7-5.4)<br>6.0% (320)	5.1 (4.2-6.4)<br>15.7% (434)	<0.001<br><0.001
FIB-4<br>FIB-4 ≥ 1.30<br>FIB-4 ≥ 2.67	0.91 (0.64-1.34)<br>26.4% (1,414)<br>3.9% (209)	1.07 (0.74-1.58)<br>37.6 % (1,042)<br>7.4% (205)	<0.001<br><0.001<br><0.001

Data presented as median (IQR) or % (n). BMI, body mass index; ALT, alanine aminotransferase; AST, aspartate aminotransferase; HDL, high density lipoprotein; LDL, low density lipoprotein; HbA1C, Hemoglobin A1C; SWE, shearwave elastography; FIB-4, fibrosis 4 variable score.

Table S7: Characteristics of the Calgary MASLD clinic patients according to age ≥65

Characteristic	Patients ≥ 65 years<br>N=1,675, 20.6%	Patients <65 years<br>N=6,451 , 79.4%	P value
Female sex	52.8% (884)	52.5% (3,377)	0.792
BMI (Kg/Height in meter^2)	30.6 (27.2-34.6)	31.4 (27.6-36.0)	<0.001
Baseline investigations			
ALT, U/L	31 (21-46)	41 (26-64)	<0.001
AST, U/L	26 (20-37)	28 (21-40)	<0.001
Albumin, g/L	38 (36-40)	39 (37-42)	<0.001
Alkaline Phosphatase, U/L	78 (64-96)	77 (64-95)	0.831
Platelets, 10E^9/L	228 (187-271)	257 (217-302)	<0.001
Triglycerides, mmol/L	1.6 (1.2-2.3)	1.8 (1.3-2.6)	<0.001
HDL, mmol/L	1.2 (1.0-1.5)	1.1 (1.0-1.4)	<0.001
LDL, mmol/L	2.2 (1.6-2.9)	2.7 (2.1-3.3)	<0.001
Total bilirubin, μmol/L	9 (6-12)	8 (6-11)	<0.001
HbA1C, %	6.0 (5.6-6.6)	5.7 (5.4-6.1)	<0.001
Diabetes mellitus	47.8% (800)	30.6% (1,972)	<0.001
Comorbidities, Charlson Index			
0	18.8% (314)	38.4% (2,477)	<0.001
1	17.9% (299)	22.8% (1,470)	
≥ 2	63.4% (1,062)	38.8% (2,504)	
SWE, valid measurements in kPa	4.9 (4.1-6.3)	4.5 (3.8-5.6)	<0.001
SWE ≥ 8 kPa	14.9% (249)	7.8% (505)	<0.001
FIB-4	1.51 (1.15-2.08)	0.86 (0.61-1.21)	<0.001
FIB-4 ≥ 1.30	65.3% (1,093)	21.1% (1,363)	<0.001
FIB-4 ≥ 2.65	13.4% (225)	2.9% (189)	<0.001

Data presented as median (IQR) or % (n). BMI, body mass index; ALT, alanine aminotransferase; AST, aspartate aminotransferase; HDL, high density lipoprotein; LDL, low density lipoprotein; HbA1C, Hemoglobin A1C; SWE, shearwave elastography; FIB-4, fibrosis 4 variable score.
