# Supplementary material for: Diabetes and obesity reduce FIB-4 accuracy in MASLD referral pathways
Source: JHEP Rep. 2026 Jan 12;8(4):101735. doi: 10.1016/j.jhepr.2026.101735 (PMC12969413; doi:10.1016/j.jhepr.2026.101735)
Supplement: Multimedia component 4 [file mmc4.pdf]

Research article

JHEP|Reports

Diabetes and obesity reduce FIB-4 accuracy in MASLD referral pathways

Authors

Abdel-Aziz Shaheen, Elizabeth Baguley, Mark G. Swain, …, Frank Lee, Alexandra Medellin, Juan G. Abraldes

Correspondence

az.shaheen@ucalgary.ca (A.-A. Shaheen), juan.g.abraldes@ualberta.ca (J.G. Abraldes).

Graphical abstract

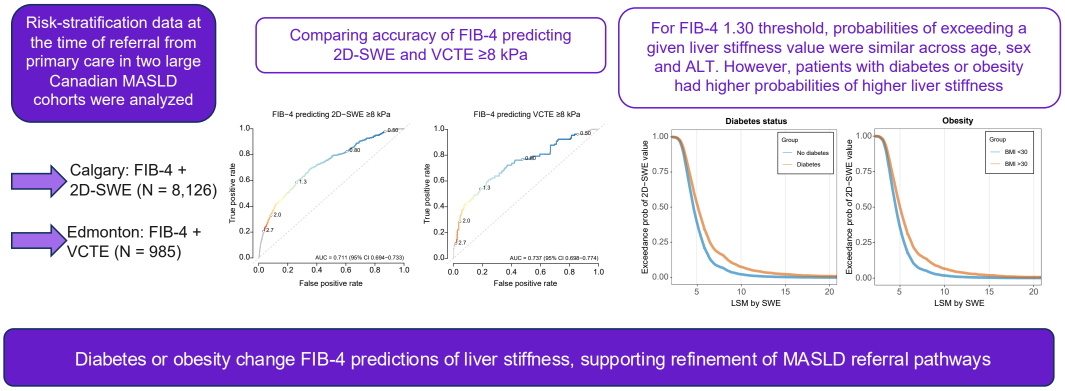

FIB-4, Fibrosis-4 index; 2D-SWE, two-dimensional shear-wave elastography; AUC, area under the curve; VCTE, vibration-controlled transient elastography; LSM, liver stiffness measurement

Highlights:

- In patients with MASLD, FIB-4 under-triages those with diabetes or obesity.
- Diabetes/BMI ≥30 increase the risk of ≥8 kPa stiffness even at FIB-4 <1.30.
- BMI and diabetes strongly modify FIB-4 performance; sex, ALT, and age have smaller effects.
- 2D-SWE thresholds align with VCTE cut-offs, supporting harmonized elastography use.

Impact and implications:

Recent guidelines recommend clinical care pathways for risk stratification of patients with MASLD (metabolic dysfunction-associated steatotic liver disease) using sensitive serum-based markers (*e.g*. fibrosis 4 index [FIB-4]) as a first step, with a cut-off of <1.30 to rule out advanced fibrosis. In patients with diabetes or obesity, the interpretation of a FIB-4 threshold of <1.30 is different than in patients without these conditions. This finding suggests the need to refine referral pathways in which FIB-4 is used as a first test. New referral models incorporating patient characteristics could improve risk stratification of patients with MASLD who need specialized liver care.

https://doi.org/10.1016/j.jhepr.2026.101735
© 2026 The Authors. Published by Elsevier B.V. on behalf of European Association for the Study of the Liver (EASL). This is an open access article under the CC BY license (http://creativecommons.org/licenses/by/4.0/). JHEP Reports, 2026, 8, 1–9

Diabetes and obesity reduce FIB-4 accuracy in MASLD referral pathways

Abdel-Aziz Shaheen[1,2,*], Elizabeth Baguley[1,2], Mark G. Swain[1], Matthew Tam[3], Mang Ming Ma[3], Giada Sebastiani[4], Jason Jiang[5], Frank Lee[5], Alexandra Medellin[6], Juan G. Abraldes[3,*]

JHEP Reports **2026**. vol. 8 | 1–9

Check for updates

Background & Aims: Primary care referral pathways often use FIB-4 to triage metabolic dysfunction-associated steatotic liver disease (MASLD), but its accuracy may vary by patient characteristics. We aimed to evaluate FIB-4 performance against elastography, compare it with other non-invasive tests, assess effect modification by BMI, diabetes, sex, ALT, and age, and calibrate 2D shear-wave elastography (2D-SWE) and vibration-controlled transient elastography (VCTE) thresholds.

Methods: We analyzed two cohorts with paired serum scores and elastography: Calgary (2D-SWE; N = 8,126) and Edmonton (VCTE; N = 985). We summarized fibrosis-risk distributions and used exceedance probabilities to align 2D-SWE with guideline-recommended VCTE cut-offs.

Results: In the Calgary cohort, median 2D-SWE was 4.6 kPa (IQR 3.9–5.8) and 9% had liver stiffness ≥8 kPa; FIB-4 was <1.30 in 70% and ≥2.67 in 5%. In the Edmonton cohort, median VCTE was 5.3 kPa (4.4–6.6) and 14% had liver stiffness ≥8 kPa; FIB-4 was <1.30 in 77% and ≥2.67 in 3%. In exceedance models, at a FIB-4 of 1.30, the probability of liver stiffness ≥8 kPa was significantly higher in individuals with diabetes or BMI ≥30 kg/m^2 than in those without these risk factors. Regression analyses showed that BMI and diabetes materially increased the probability of liver stiffness ≥8 kPa at a given FIB-4, whereas sex, elevated ALT, and age had smaller effects. Using the recommended 2D-SWE thresholds of 9, 13, 17 kPa, we observed an expected stepwise correspondence with VCTE 15, 20, 25 kPa, supporting harmonised elastography cut-offs across modalities.

Conclusion: FIB-4 alone under-triages patients with diabetes and/or obesity. These patients should be fast-tracked to elastography even when FIB-4 is <1.30. We recommend validating these findings in other cohorts, as they may affect triaging practices.

© 2026 The Authors. Published by Elsevier B.V. on behalf of European Association for the Study of the Liver (EASL). This is an open access article under the CC BY license (http://creativecommons.org/licenses/by/4.0/).

Introduction

The prevalence of metabolic dysfunction-associated steatotic liver disease (MASLD) has significantly increased over the last two decades, with more than 30% of the population worldwide reported to be affected.[1,2] Patients with MASLD and advanced liver fibrosis are more likely to have liver-related causes of morbidity and mortality compared to those with lesser degrees of fibrosis.[3] Moreover, advanced liver fibrosis is associated with higher cardiovascular disease-related morbidity and mortality.[4]

Recent guidelines recommend clinical care pathways for risk-stratification of patients with MASLD using serum-based markers (*e.g.* fibrosis-4 index [FIB-4]) with high negative predictive value (NPV) cut-offs as a first step.[5,6] The main goal of MASLD clinical pathways is to use simple serum-based markers first, followed by radiological modalities (*e.g.* vibration-controlled transient elastography [VCTE] or two-dimensional shear wave elastography [2D-SWE]) or specific serum-based markers (such as enhanced liver fibrosis [ELF]) to identify patients at-risk of advanced fibrosis who may benefit from further evaluation, management and treatment.[5,7,8] Indeed, guidelines for the use of the recently approved treatment for MASLD (resmetirom) propose elastography-based decision rules to initiate treatment, making elastography the new standard in clinical practice to risk stratify patients with MASLD.[9,10]

As FIB-4 values increase with older age, it is recommended to use different FIB-4 cut-offs according to age, with a FIB-4 cut-off of ≥1.3 for patients aged <65 years and ≥2.0 for those aged ≥65 years.[11] While patients with diabetes mellitus have a higher prevalence of advanced fibrosis, current recommendations are to use the same FIB-4 cut-offs for hepatology referral, and second-line assessment with liver stiffness measurement (LSM) regardless of diabetes status.[5,11] Previous studies showed poor accuracy of FIB-4 to identify advanced fibrosis among patients with diabetes, and FIB-4 accuracy was dependent on BMI among those patients.[12,13] Therefore, in this study, we used data from two large cohorts of patients with MASLD who were initially evaluated in primary care and underwent risk stratification with both FIB-4 and LSM (by 2D-

* Corresponding authors. Address: Room 6D25, 3280 Hospital Drive NW, Calgary, Alberta, T2N 4Z6, Canada; Tel: 403-592-5034, Fax: 403-592-5090.
E-mail addresses: az.shaheen@ucalgary.ca (A.-A. Shaheen), juan.g.abraldes@ualberta.ca (J.G. Abraldes).
https://doi.org/10.1016/j.jhepr.2026.101735

SWE or VCTE) to understand the impact of patient characteristics on the association between FIB-4 and LSM. Our primary objective was to evaluate the performance of FIB-4 as an initial risk stratification tool in a large primary care cohort of patients with risk factors for MASLD, considering key clinical factors such as age, sex, liver enzymes, BMI, and diabetes mellitus, using 2D-SWE or VCTE as reference tests. Our secondary objective was to develop a predictive model integrating FIB-4 and these clinical variables to estimate different LSM thresholds with potential therapeutic relevance.

Patients and methods

Study setting and design

In this retrospective study, we included participants evaluated through the first phase of Calgary MASLD pathway implementation between 2017 and 2022; and the Edmonton MASLD pathway between 2016 and 2021. The development and performance of the Calgary and Edmonton clinical care pathways have been previously published.[14,15] The Calgary pathway serves primary care providers (PCPs) in the Calgary Health Zone (population of ~1.6 million) providing them with a framework to directly assess patients with a history of metabolic syndrome (*e.g*. diabetes, dyslipidemia or obesity) or evidence of steatotic liver on prior imaging for 2D-SWE assessment provided through community-based radiology providers. PCPs were expected to complete a full workup to rule out other causes of chronic liver disease, such as metabolic dysfunction- and alcohol-related liver disease (MetALD) and viral hepatitis, by following a pathway-outlined stepwise algorithm. 2D-SWE was chosen as the first step in MASLD assessment because, in preliminary discussions about pathway design and implementation, PCPs preferred 2D-SWE over FIB-4. This preference was due to a simpler referral process, reduced reliance on calculating FIB-4 in busy primary care settings, and the availability of radiology providers using a single validated 2D-SWE platform (Aplio i800; Canon Medical Systems).[14,16] Patients with 2D-SWE ≥8.0 kPa, or inconclusive results, were referred to a single MASLD hepatology clinic in Calgary. The Edmonton primary care MASLD pathway had different referral criteria: abnormal transaminases in the context of metabolic syndrome or a finding of steatosis on prior imaging. During the period between 2016 and 2021 all patients had FIB-4 and VCTE regardless of FIB-4 values.

Study population

During the study period, 12,122 adult individuals were assessed by 2D-SWE in the Calgary health zone by primary care. As PCPs did not consistently order aspartate aminotransferase (AST) to calculate FIB-4 at the time of 2D-SWE assessment, 8,590 patients had an available AST value to calculate FIB-4 within 6 months of 2D-SWE assessment. Patients excluded from the study cohort included those consuming more than 10 standard alcoholic drinks per week for women, or 15 standard drinks per week for men (n = 21), and pediatric patients (n = 1). Consistent with current nomenclature, our cohort includes patients with MASLD and MetALD. Patients with inconclusive 2D-SWE (n = 374) were excluded. Therefore, 8,126 patients were included in this study. Similar exclusion criteria were implemented in the Edmonton cohort and there were 985 patients eligible for this study with available FIB-4 and valid VCTE. Inclusion and exclusion criteria for our MASLD cohorts are shown in Fig. S1.

Data sources

We used the Calgary and Edmonton MASLD pathway databases, which include 2D-SWE and VCTE radiology data; the Alberta provincial laboratory database, capturing ≥97% of all laboratory investigations for Alberta residents; and Alberta administrative databases (inpatient, outpatient, and physician claims).

Outcomes and main variables of interest

The study had two outcomes: (a) estimation of the probability of exceeding LSM thresholds by 2D-SWE or VCTE across FIB-4 values; and (b) assessment of the performance of FIB-4 in predicting LSM according to age, sex, BMI, transaminases, and diabetes status. The main variables of interest were: a) biological sex; b) diabetes mellitus defined as: Hemoglobin A1c (HbA1c) >6.4%, a diagnosis code of diabetes by a physician, or patients using diabetes-related medications and a history of HbA1c >6.4%; and c) BMI (weight in kg/m^2). We also created two obesity measurements separating class 1 *vs*. class 2/3 (dichotomous BMI: <35 and ≥35) or living with obesity *vs*. not (BMI: <30 and >30) to understand the impact of obesity on study outcomes. We followed this classification as less than 5% of our cohort were of Asian ethnicity. We defined normal alanine aminotransferase (ALT) level at baseline as <30 U/L for men, and <25 U/L for women.[17] We compared FIB-4 with other non-invasive tests (NITs) for fibrosis, including LiverPRO,[18] and the fibrotic NASH index (FNI)[8] scores against elastography-defined elevated stiffness: 2D-SWE ≥8 kPa (Calgary) and VCTE ≥8 kPa (Edmonton). Pairwise tests used pairwise complete cases. ROC/AUROC were estimated non-parametrically with bootstrap 95% CIs (100 replicates), and pairwise AUC differences were tested by DeLong (two-sided α = 0.05).[19]

Covariates

Laboratory data were obtained at baseline (within 6 months of 2D-SWE assessment). Serum ALT, AST, albumin, alkaline phosphatase, gamma glutamyltransferase, platelets, triglycerides, high-density lipoprotein, low-density lipoprotein, total bilirubin, international normalized ratio, creatinine, and HbA1c were collected. We documented the presence of comorbidities using the Charlson comorbidity index[20] defined using categorical variables (0, 1 or ≥2 comorbidities). Moderate or severe liver disease comorbidities as well as diabetes mellitus components were removed from the Charlson comorbidity index.

Statistical analyses

Patient characteristics were described as median (IQR) or percentage (n) for the MASLD pathway primary care-based cohort. Chi squared (X^2) test and Wilcoxon rank-sum test were used to study differences between our two study cohorts according to our main exposure variables. Cut-off points for LSM determined by 2D-SWE and used to define "high risk" MASLD are arbitrary, and there is ongoing discussion around the threshold of LSM by 2D-SWE that should lead to

hepatology referral or to treatment initiation. To model the associations between FIB-4 and 2D-SWE, and the effect of the modifying variables, we used ordinal regression for a continuous outcome (in this case LSM by 2D-SWE) (with the orm function in the rms package, R software). In this way we avoided assuming beforehand a definite target threshold of 2D-SWE.[21] This model allows calculation of exceedance probabilities for every potential 2D-SWE threshold, according to values of the predictors. Details of the modeling process and illustrative examples are provided in the supplementary methods.[22]

To establish an indirect comparison of VCTE and 2D-SWE values, we modeled the association between FIB-4 and VCTE with ordinal regression in the Edmonton sample, and between FIB-4 and 2D-SWE in a subsample of the Calgary cohort, matched 1:1 with the Edmonton cohort by age, sex, presence of diabetes, BMI and abnormal transaminases. We used nearest neighbor matching without replacement, with a 1:1 matching ratio to pair each participant in the Calgary cohort with the closest match in the Edmonton cohort based on a logistic regression score. Matching was performed with the MatchIt package in R, specifying method = "nearest" and distance = "logit" for the matching algorithm.[23] Results of the matching are shown in the supplementary methods. This approach, by anchoring both cohorts on FIB-4, allows an indirect comparison of VCTE and 2D-SWE thresholds. To assess potential selection bias related to missing AST (and therefore missing FIB-4), we compared baseline demographics, laboratory values, comorbidities, and 2D-SWE between patients with and without an available FIB-4. We also conducted a sensitivity analysis using BMI ≥30 kg/m^2 in addition to our primary stratification at BMI ≥35 kg/m^2. Analyses were performed using Stata IC (version 17.1, Texas, USA) and R version 4.3.2 (R Core Team, Vienna, Austria). The study protocol was reviewed and approved by the Research Ethics Committee at the University of Calgary, Calgary, Alberta (REB17-2142).

Results

MASLD pathway cohort characteristics

Patient characteristics of the Calgary and Edmonton MASLD primary care cohorts are shown in Table 1. In the Calgary MASLD cohort, the median age was 54 years (IQR 43-63) and 53% of patients were female. Obesity (median BMI 31.2 [IQR 27.6-35.7]) and diabetes (34%) were the main indications for enrollment in the MASLD pathway by primary care. The median ALT was 38 U/L (IQR 25-60), with 5,720 (70%) patients in the Calgary cohort having an elevated ALT level. In contrast, patients in the Edmonton cohort were younger (median age 43 [35-53]), more likely to be male (67%), and most had elevated ALT and AST levels. Characteristics of patients with FIB-4 ≥1.30 or 2D-SWE ≥8 kPa in the Calgary cohort are shown in Table 1.

Comparing Calgary patients with available FIB-4 data (N = 8,126) to those without (n = 3,532), age (54 *vs.* 55 years) and BMI (31.2 *vs.* 31.5 kg/m^2) were similar. Patients with FIB-4 showed higher female representation (53% *vs.* 49%) and slightly higher diabetes prevalence (34% *vs.* 32%). Median 2D-SWE values were similar between groups (4.6 *vs*. 4.5 kPa), indicating no difference in disease severity by FIB-4 availability (Table S1).

Performance of FIB-4 and other NITs compared to 2D-SWE and VCTE in the primary care MASLD cohorts

We first assessed the performance of FIB-4 in risk stratifying our MASLD cohorts using recommended cut-offs. In the Calgary MASLD cohort, FIB-4 classified 5% as ≥2.67, 25%

Table 1. Patient characteristics.

Characteristic	Calgary MASLD cohort N = 8,126	Calgary MASLD patients with FIB-4 ≥1.30 n = 2,476	Calgary MASLD patients with 2D-SWE ≥8 kPa n = 754	Edmonton MASLD cohort N = 985
Age, yrs.	54 (43-63)	63 (56-69)	60 (51-67)	43 (35-53)
Female sex	52.5% (4,261)	48.5% (1,199)	53.5% (402)	32.6% (321)
BMI (kg/m^2)	31.2 (27.6-35.7)	30.9 (27.3-35.2)	34.6 (29.9-40.4)	30.9 (27.7-35.1)
Baseline investigations				
ALT, U/L	38 (25-60)	38 (25-61)	40 (26-66)	61 (42-87)
AST, U/L	28 (21-40)	38 (27-56)	36 (23-55)	36 (28-49)
Albumin, G/L	39 (37-41)	39 (37-41)	38 (36-40)	45 (43-47)
ALP, U/L	77 (64-95)	78 (64-99)	86 (69-108)	80 (67-97)
Platelets, 10E^9/L	251 (210-297)	205 (172-241)	221 (176-273)	236 (199-278)
Triglycerides, mmol/L	1.8 (1.2-2.5)	1.6 (1.1-2.3)	1.7 (1.2-2.4)	1.7 (1.2-2.3)
HDL, mmol/L	1.2 (1.0-1.4)	1.2 (1.0-1.5)	1.1 (0.9-1.3)	1.1 (1.0-1.3)
LDL, mmol/L	2.6 (1.9-3.3)	2.3 (1.7-3.0)	2.2 (1.6-2.9)	2.9 (2.3-3.5)
Total bilirubin, µmol/L	8 (6-11)	9 (7-13)	9 (6-13)	11 (9-15)
HbA1c, %	5.8 (5.5-6.2)	5.8 (5.5-6.5)	6.1 (5.6-7.1)	5.7 (5.4-6.1)
Diabetes mellitus	34.1% (2,772)	42.5% (1,052)	57.6% (434)	22.1% (218)
Comorbidities, Charlson Index				
0	34.4% (2,791)	24.5% (607)	18.2% (137)	
1	21.7% (1,769)	19.5% (482)	15.0% (113)	
≥2	43.9% (3,566)	56.0% (1,387)	66.8% (504)	
2D-SWE or VCTE, valid measurements in kPa	4.6 (3.8-5.7)	5.2 (4.2-6.7)		5.3 (4.4-6.6)
2D-SWE ≥8 kPa	9.3% (754)	18.0% (442)		13.6% (134)
FIB-4	0.97 (0.67-1.42)		1.48 (0.98-2.42)	0.84 (0.61-1.26)
FIB-4 ≥1.30	30.4% (2,476)		58.6% (442)	23.5% (231)
FIB-4 ≥2.67	5.1% (414)		20.8% (157)	3.2% (31)

Data presented as median (IQR) or % (n). 2D-SWE, two-dimensional shear wave elastography; ALP, alkaline phosphatase; ALT, alanine aminotransferase; AST, aspartate aminotransferase; FIB-4, fibrosis 4 index; HbA1c, hemoglobin A1C; VCTE, vibration-controlled transient elastography.

Table 2. Association between 2D-SWE, VCTE and FIB-4 per groups of interest in Calgary and Edmonton cohorts.

Groups of interest	FIB-4 categories	Calgary cohort 2D-SWE ≥8 kPa	Calgary cohort 2D-SWE ≥6.5 kPa	Edmonton cohort VCTE ≥8 kPa
Main cohort (Calgary = 8,126) (Edmonton = 985)	<1.30	5.5% (312/5,670)	10.7% (604/5,670)	8.1% (61/754)
	1.30-2.66	14.0% (285/2,042)	23.4% (477/2,042)	27.0% (54/200)
	≥2.67	37.9% (157/414)	47.8% (198/414)	61.3% (19/31)
Patients with elevated ALT (Calgary = 5,720) (Edmonton = 907)	<1.30	5.0% (200/3,997)	10.6% (422/3,997)	8.6% (60/702)
	1.30-2.66	15.6% (217/1,394)	25.8% (360/1,394)	28.8% (51/177)
	≥2.67	40.7% (134/329)	51.1% (168/329)	64.3% (18/28)
Patients with normal ALT (Calgary = 2,406) (Edmonton = 78)	<1.30	6.7% (112/1,673)	10.9% (182/1,673)	1.9% (1/52)
	1.30-2.66	10.5% (68/648)	18.1% (117/648)	13.0% (3/23)
	≥2.67	27.1% (23/85)	35.3% (30/85)	33.3% (1/3)
Patients with DM (Calgary = 2,772) (Edmonton = 218)	<1.30	9.3% (160/1,730)	16.1% (278/1,730)	17.8% (24/135)
	1.30-2.66	21.0% (176/837)	31.9% (267/837)	47.1% (32/68)
	≥2.67	47.8% (98/205)	57.6% (118/205)	80.0% (12/15)
Patients without DM (Calgary = 5,354) (Edmonton = 767)	<1.30	3.9% (152/3,940)	8.3% (326/3,940)	6.0% (37/619)
	1.30-2.66	9.1% (109/1,205)	17.4% (210/1,205)	16.7% (22/132)
	≥2.67	28.2% (59/209)	38.3% (80/209)	43.8% (7/16)
Female patients (Calgary = 4,261) (Edmonton = 321)	<1.30	6.2% (191/3,070)	11.4% (349/3,070)	9.3% (20/216)
	1.30-2.66	12.6% (124/982)	20.7% (203/982)	34.1% (31/91)
	≥2.67	41.6% (87/209)	52.2% (109/209)	78.6% (11/14)
Male patients (Calgary = 3,852) (Edmonton = 664)	<1.30	4.6% (120/2,591)	9.8% (254/2,591)	7.6% (41/538)
	1.30-2.66	15.2% (160/1,056)	25.8% (272/1,056)	21.1% (23/109)
	≥2.67	34.2% (70/205)	43.4% (89/205)	47.1% (8/17)
Patients with BMI <35 (Calgary = 4,699) (Edmonton = 735)	<1.30	3.3% (108/3,253)	6.6% (216/3,253)	4.6% (26/569)
	1.30-2.66	9.2% (110/1,193)	17.6% (210/1,193)	18.9% (27/143)
	≥2.67	32.8% (83/253)	44.7% (113/253)	65.2% (15/23)
Patients with BMI ≥35 (Calgary = 1,845) (Edmonton = 250)	<1.30	10.9% (146/1,339)	19.7% (264/1,339)	18.9% (35/185)
	1.30-2.66	22.8% (98/430)	33.7% (145/430)	47.4% (27/57)
	≥2.67	47.4% (36/76)	52.6% (40/76)	50.0% (4/8)
Patient age ≥65 years (Calgary = 1,675) (Edmonton = 68)	<1.30	7.0% (41/582)	12.0% (70/582)	0% (0/8)
	1.30-2.66	13.8% (120/868)	23.2% (201/868)	29.6% (13/44)
	≥2.67	39.1% (88/225)	49.3% (111/225)	62.5% (10/16)
Patient age <65 years (Calgary = 6,451) (Edmonton = 917)	<1.30	5.3% (271/5,088)	10.5% (534/5,088)	8.2% (61/746)
	1.30-2.66	14.1% (165/1,174)	23.5% (276/1,174)	26.3% (41/156)
	≥2.67	36.5% (69/189)	46.0% (87/189)	60.0% (9/15)

2D-SWE, two-dimensional shear wave elastography; ALT, alanine aminotransferase; DM, diabetes mellitus type 2; FIB-4, fibrosis 4 variable score; VCTE, vibration-controlled transient elastography.

between 1.30 and 2.66, and 70% <1.30. In the Edmonton MASLD cohort, FIB-4 classified 3% as ≥2.67, 20% between 1.30 and 2.66, and 77% <1.30. In the Calgary cohort the median 2D-SWE LSM value was 4.6 kPa (IQR: 3.8-5.7) and 9% had 2D-SWE ≥8.0 kPa. In the Edmonton cohort, the median VCTE LSM measurement was 5.3 kPa (IQR 4.4-6.6) and 14% had VCTE ≥8.0 kPa (Table 1).

Prevalence of LSM ≥8.0 kPa measured by 2D-SWE in the Calgary MASLD cohort or VCTE in the Edmonton MASLD cohort among different categories of FIB-4 (<1.30, 1.30-2.66, and ≥2.67) are presented in Table 2. Furthermore, data on 2D-SWE ≥6.5 kPa are also presented. Overall, prevalence of 2D-SWE ≥8.0 kPa or VCTE ≥8.0 kPa was significantly higher among FIB-4 categories in patients with elevated ALT, diabetes, females, or BMI >35, p <0.001 (Table 2). However, the prevalence of 2D-SWE ≥8 kPa among patients with FIB-4 <1.30 was higher among patients with normal ALT compared to elevated ALT (7% *vs.* 5%, p = 0.013); 2D-SWE ≥8.0 kPa ratios were similar between males and females in patients with FIB-4 values between 1.30 and 2.67 (15% *vs.* 13%, p = 0.110); and VCTE ≥8.0 kPa was similar between patients with BMI <35 and BMI ≥35 in patients with FIB-4 values ≥2.67 (63% *vs.* 50%, p = 0.761). Findings were directionally consistent when obesity was defined as BMI ≥30 kg/m^2; estimates and contrasts were similar (Table S5). Interestingly, ratios of 2D-SWE or VCTE LSM measurements ≥8.0 kPa were two times higher among patients with diabetes than in patients without diabetes in all FIB-4 categories. In Calgary, AUROCs were FIB-4 0.709 (95% CI 0.689–0.728), LiverPRO 0.718 (0.701–0.736), and FNI 0.669 (0.648–0.689). FIB-4 *vs.* LiverPRO p = 0.433, FIB-4 *vs.* FNI p <0.001, LiverPRO *vs.* FNI p <0.001. In Edmonton, AUROCs were FNI 0.793 (0.743–0.833), FIB-4 0.737 (0.698–0.774), and LiverPRO 0.718 (0.673–0.756). FNI was more accurate compared to FIB-4 p = 0.023 and LiverPRO p = 0.008. FIB-4 performed similarly to LiverPRO p = 0.372 (Fig. 1).

Characteristics of the calgary MASLD cohort according to variables of interest

In the Calgary MASLD cohort, female patients were older, had higher BMI, and were more likely to have diabetes and comorbidities compared to males (Table S2). Patients with normal ALT were older, more likely female, and more likely to have ≥2 comorbidities compared to patients with elevated ALT (Table S3). Class 2/3 obesity (BMI ≥35 kg/m^2) was prevalent in the Calgary MASLD pathway cohort (28%). Patients with BMI ≥35 were more likely to be younger, female, and have diabetes and comorbidities (Table S4). Patients with MASLD and diabetes were more likely to be older, female, and have higher BMI and more comorbidities (Table S5). Patients aged >65 years had lower BMI compared to patients aged <65 years and were more likely to have diabetes and comorbidities (Table S6). Laboratory data differences according to exposure variables are shown in the supplementary tables.

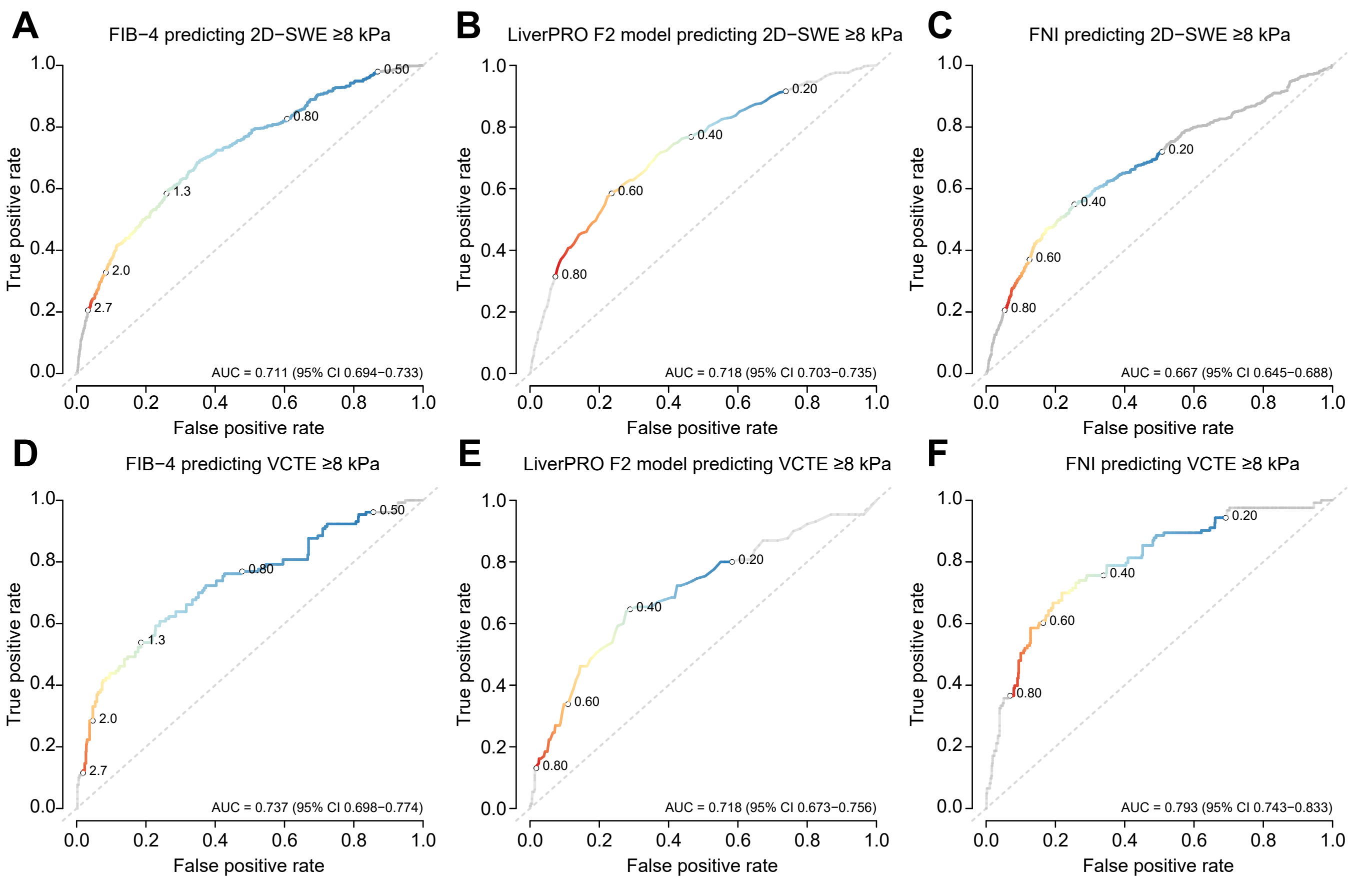

Fig. 1. Receiver-operating characteristic curves showing the discriminative value of non-invasive tests for detecting 2D-SWE ≥8 or VCTE ≥8. Receiver-operating characteristic curves showing the discriminative value of FIB-4, LiverPRO and FNI for detecting 2D-SWE ≥8 (A-C) or VCTE ≥8 (D-F). 2D-SWE, two-dimensional shear wave elastography; FIB-4, fibrosis-4 index; FNI, fibrotic NASH index; VCTE, vibration-controlled transient elastography.

Association between FIB-4 and 2D-SWE according to patient characteristics

Since the 2D-SWE threshold for referral to hepatology might evolve according to available treatments, guideline recommendations, and available resources, we modelled the association of FIB-4 values with the exceedance probabilities of any value of 2D-SWE, testing the influence of patient characteristics on FIB-4 predictions. As shown in Fig. 1, for a given FIB-4 value, patients with diabetes, BMI >35, and abnormal transaminases had a greater probability of having higher 2D-SWE values. On the other hand, age and sex did not have a major impact on FIB-4 predictions. As shown in Fig. 2, using a FIB-4 cut-off threshold of 1.30, there is risk of missing patients with 2D-SWE ≥8 kPa; however, the risk of missing high 2D-SWE values (*i.e.* >12 kPa) is very low, even in patients with unfavorable characteristics.

Furthermore, we developed a unified model to predict different 2D-SWE thresholds according to FIB-4 values and the five variables tested above. After backwards elimination, FIB-4, BMI, sex, diabetes and abnormal transaminases were retained in the model. Details of this model and instructions for calculating the exceedance probability of different 2D-SWE thresholds (with examples), together with a graphical representation of the model are presented in detail in the supplementary methods.

Validation in the Edmonton MASLD cohort using VCTE

The Edmonton-MASLD cohort, with paired FIB-4/VCTE values in 985 patients, had a slightly different patient profile than the Calgary-MASLD cohort. Still, there was a similar impact of the clinical variables on FIB-4 predictions as we saw with the FIB4-2D-SWE cohort. Indeed, patients with diabetes, BMI >35, and abnormal transaminases showed a higher probability of having a VCTE ≥8 kPa at FIB-4 thresholds of 1.30 and 2.67 (Table 2). However, the probability of having a VCTE ≥8 kPa was substantially higher than the probability of having a 2D-SWE ≥8 kPa in the Calgary cohort and it was closer to 2D-SWE ≥6.5 kPa (Table 2).

To determine whether this difference was driven by distinct patient profiles between cohorts or by systematic differences between the two techniques, we modeled the association between FIB-4 and VCTE in the Edmonton cohort, and between FIB-4 and 2D-SWE in a subsample of the Calgary cohort matched 1:1 to the Edmonton cohort by age, sex, diabetes status, elevated transaminases, and BMI. Figure 3 presents the graphical representation of both models, including indicators for the VCTE "rule of 5s" thresholds (10, 15, 20, 25 kPa) and the World Federation for Ultrasound in Medicine and Biology (WFUMB) "rule of four" 2D-SWE thresholds (5, 9, 13, 17 kPa).[22] The 15, 20, and 25 kPa VCTE thresholds corresponded approximately to the 9, 13, and 17 kPa 2D-SWE thresholds.

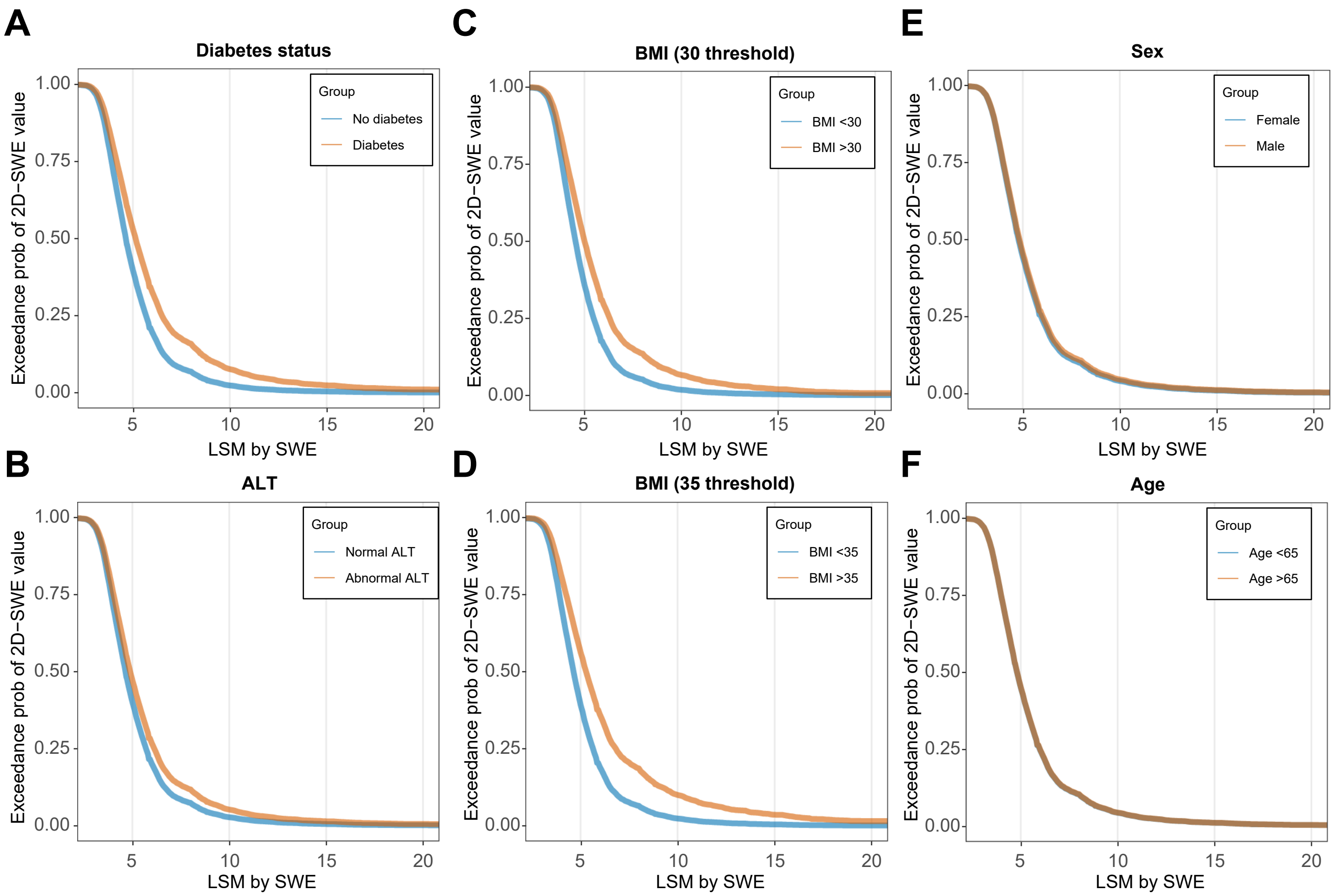

Fig. 2. Exceedance probabilities of 2D-SWE for a FIB-4 value of 1.3. Each panel shows the exceedance probability, the probability that a patient's 2D-SWE will be at or above the value on the x-axis (kPa). Curves compare two strata of the covariate named in each panel (A: diabetes; B: abnormal ALT; C: BMI with a 30 threshold; D: BMI with a 35 threshold; E: sex; F: age strata as shown in the panel legends). Higher curves indicate a greater chance of exceeding any given 2D-SWE threshold for the given 1.3 FIB-4 value. Estimates come from ordinal regression models (introducing one variable at a time) as explained in the methods and supplementary methods section. These findings suggest that a FIB-4 of 1.3 predicts higher values of 2D-SWE in individuals with diabetes, obesity and abnormal ALT, whereas the effect of age and sex is minor. 2D-SWE, shear-wave elastography; ALT, alanine aminotransferase; FIB-4, fibrosis-4 index.

Additionally, the 8 and 12 kPa VCTE thresholds corresponded approximately to 6.5 and 8 kPa 2D-SWE, respectively.

Discussion

In this study, we describe the impact of clinical characteristics on current recommendations for risk-stratification of patients with MASLD. We evaluated two large cohorts identified from primary care-based MASLD pathways in Canada. To our knowledge, our study reports findings from two of the largest MASLD cohorts evaluated within primary care.[6] Our cohort characteristics were different in terms of age, sex distribution and indications for MASLD risk stratification. However, they were comparable to other MASLD populations.[24–26] In our study, we addressed clinical questions on the impact of certain patient demographic and clinical characteristics that may affect performance of recommended MASLD clinical care pathways. In both of our cohorts, we observed that patients with obesity and diabetes have a much higher proportion of high elastography values for a given FIB-4 value.

A distinct aspect of our study is the inclusion of data from the Calgary MASLD pathway, which includes patients with metabolic risk factors beyond elevated transaminases. We reported risk stratification performance among patients with normal liver enzymes and class 2/3 obesity that were not available in previous clinical care pathways. Specifically, clinical care pathways that have been implemented worldwide to risk-stratify patients with MASLD in primary care have focused on evaluating patients who have either elevated ALT levels[27,28] or metabolic dysfunction as a risk factor for MASLD.[14,29–31] These pathways used FIB-4, NAFLD fibrosis score, or the AST/ALT ratio as an initial step in patient risk stratification, followed by VCTE or ELF as a second test to confirm the presence of significant liver fibrosis. The observed prevalence of ≥8 kPa in our cohorts is aligned with risk-enriched clinical pathways, consistent with our cohort composition and referral strategy.[32,33]

Head-to-head comparisons of recent NITs showed cohort-specific performance. In Calgary, FIB-4 and LiverPRO had similar AUROC accuracy and exceeded FNI. However, in Edmonton, FNI outperformed FIB-4 and LiverPRO. We therefore retain FIB-4 as the default for simplicity and availability. International guidelines recommend using FIB-4 as an initial step for risk stratification. In our cohorts, ~23-30% of patients with MASLD had a FIB-4 score ≥1.30, similar to previous

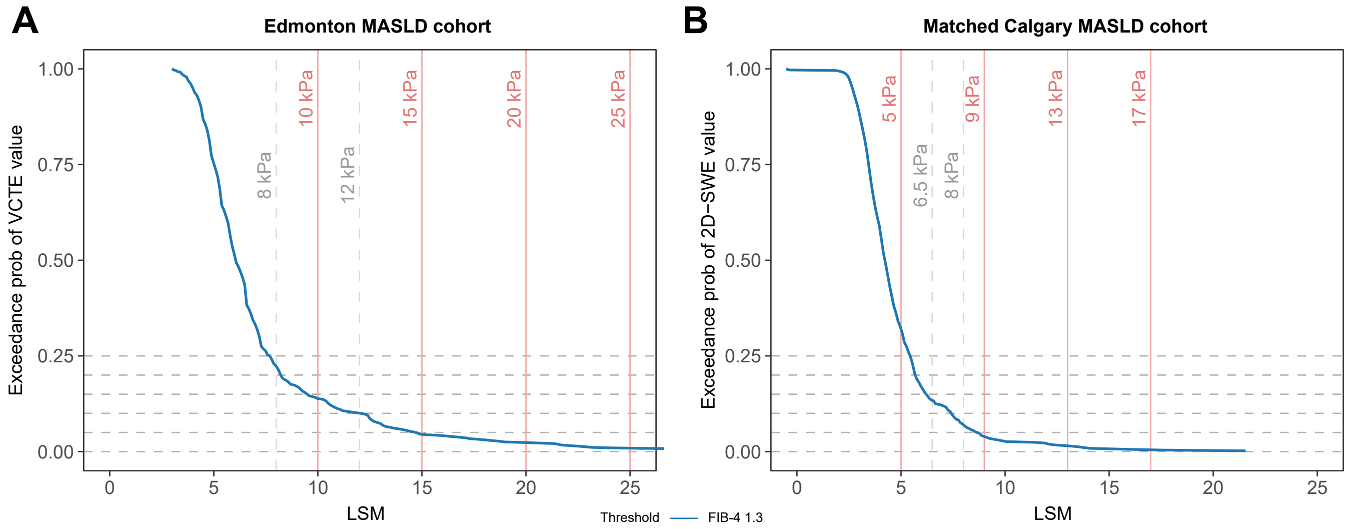

Fig. 3. Exceedance probabilities of LSM for a FIB-4 value of 1.3. (A) VCTE in the Edmonton cohort. (B) 2D-SWE in the matched Calgary cohort. Horizontal dashed lines indicate reference probabilities (5%, 10%, 15%, 20%, and 25%). Vertical red lines represent the "rule of 5s" for VCTE, and the "rule of 4s" for 2D-SWE. Additional grey lines represent the 8 and 12 kPa VCTE threshold, and how these approximate 6.5 and 8 kPa thresholds for 2D-SWE in the matched Calgary sample. The figure illustrates that the same FIB-4 value of 1.3 predicts higher VCTE values than 2D-SWE values. For example, whereas a FIB-4 of 1.3 is associated with a 4% probability of finding a VCTE value ≥15 kPa in the Edmonton cohort, it is associated with a 4% probability of finding an 2D-SWE value ≥9 kPa in the Calgary cohort (see Results for details). As shown in the plot, the 9, 13, 17 values of the "rule of 4s" for SWE, might reflect the 15, 20 and 25 kPa values of the rule of 5s in VCTE. 2D-SWE, two-dimensional shear wave elastography; FIB-4, fibrosis-4 index; LSM, liver stiffness measurement; VCTE, vibration-controlled transient elastography.

findings in other pathways.[6,27] As reported by Pierce *et al.*, there is significant variability between 2D-SWE machines, but each 2D-SWE system demonstrates reasonable reliability.[34] Therefore, we used a single 2D-SWE machine in the Calgary cohort.

Our findings show how WFUMB 'rule of four' thresholds map onto commonly used VCTE cut-offs in MASLD referral pathways. In our data, 2D-SWE values of 9–13–17 kPa aligned in an expected stepwise fashion with VCTE thresholds of 15-20 and 25 kPa, supporting the use of established WFUMB thresholds to harmonize elastography-based triage across modalities. To validate these findings, we matched both cohorts (Calgary and Edmonton 1:1 by age, sex, diabetes, abnormal transaminases and BMI) and preformed an indirect comparison of 2D-SWE and VCTE by anchoring both values on FIB-4, assuming that in the matched cohorts, FIB-4 predictions should be roughly equivalent. In the matched Calgary cohort, a FIB-4 of 1.30 yielded a 10% exceedance probability of having a 2D-SWE ≥8 kPa, whereas in the Edmonton cohort, it gave a 10% exceedance probability of having a VCTE ≥12 kPa. This indirect comparison suggests that a 2D-SWE threshold of 8 kPa could be roughly equivalent to a VCTE threshold of 12 kPa. These findings confirm previous reports that clinically relevant thresholds for 2D-SWE are lower than with VCTE, and refinement of decision thresholds is essential in implementing 2D-SWE-based risk-stratification on a large scale.[11,22]

In our study, we evaluated the influence of sex and age on the performance of FIB-4 followed by LSM measured using either 2D-SWE or VCTE. Although MASLD is more prevalent among males, females more commonly have advanced fibrosis, particularly after menopause.[35] Our cohorts differed in sex distribution; however, LSM values measured by both 2D-SWE and VCTE were higher in females across all FIB-4 categories. Interestingly, after adjusting for BMI, diabetes and abnormal ALT, sex had only a minor predictive value and, in fact, male sex predicted higher 2D-SWE values for a given FIB-4. These findings emphasize the importance of examining sex-specific differences in non-invasive fibrosis testing and the need for sex-oriented referral pathways for MASLD.

Age is a known predictor of fibrosis progression in MASLD and other chronic liver diseases. Because older age increases FIB-4 values, current guidelines recommend using higher FIB-4 cut-offs for patients older than 65 years. Elevated LSM measured by either 2D-SWE or VCTE was similar between patients stratified by the 65-year age threshold. Furthermore, in our FIB-4 based regression models, age did not predict elevated 2D-SWE or VCTE values, consistent with our previous findings.[15]

Most patients in our MASLD cohorts had elevated ALT at baseline. However, one third of patients assessed in the Calgary MASLD cohort had normal ALT. Interestingly, patients with normal ALT had higher BMI and lower prevalence of diabetes, compared to those with elevated ALT. LSMs were higher across all categories of FIB-4 among patients with elevated ALT compared to patients with normal ALT. Therefore, in our predicting elevated LSM models, elevated transaminases were a significant predictor.

Obesity was prevalent in our cohorts, with a median BMI of 31.2 and 30.9 kg/m^2 in the Calgary and Edmonton cohorts, respectively. This distinguishes our study from many published cohorts from Europe and Asia who included patient populations with lower BMIs.[6] We show that BMI has a significant impact on the association between FIB-4 and 2D-SWE or VCTE values, questioning the use of the same FIB-4 threshold (1.30) for initial referral triage in patients with class 2/3 obesity.[36] A portion of the higher exceedance we observed in obesity class 2/3 may reflect measurement bias rather than true histologic fibrosis. Obesity increases skin-to-liver distance and alters shear-wave propagation, which can inflate

elastography values.[37] Consistent with this, some liver biopsy-based cohorts have reported inverse or no relationships between BMI and fibrosis stage.[38,39] While obesity class 2/3 is associated with higher rates of significant and advanced fibrosis measured by LSM in our cohorts, it is recommended to repeat elastography or use further tests such as magnetic resonance elastography or liver biopsy to confirm fibrosis stage among these patients.[40,41]

The recognition that diabetes is a major risk factor for developing advanced fibrosis among patients with MASLD is growing.[42,43] Similar to our study, a recent meta-analysis identified that patients with diabetes have two-times the risk of developing advanced liver fibrosis.[42] In our cohort, the proportion of patients with a FIB-4 <1.30 who had high 2D-SWE values was much higher in patients with diabetes than without diabetes. Our findings support recent recommendations calling for a fast-track or dedicated pathway to assess liver fibrosis in patients with MASLD and diabetes, who have the highest prevalence of advanced fibrosis.[44,45]

Our study has some limitations. We addressed potential selection bias from AST ordering and FIB-4 availability by explicitly comparing patients with *vs.* without FIB-4; groups were broadly similar, with near-identical median 2D-SWE. In our jurisdiction, patients with suspected alcohol-related liver disease (ALD) are routinely triaged through a dedicated ALD pathway, which likely diverts patients with ALD away from the MASLD pathway. Consequently, our primary-care cohort reflects a blended MASLD/MetALD population. We recognize that using 2D-SWE may not be feasible in other jurisdictions with limited resources, or with ultrasound providers using different types of 2D-SWE machines. To mitigate this limitation, we collaborated with a radiology group well trained in 2D-SWE and who used a single type of 2D-SWE machine. Furthermore, we used an external cohort with paired FIB-4 and VCTE that allowed us to indirectly benchmark the interpretation of 2D-SWE in relation to VCTE values. Validation of our findings using different 2D-SWE machines are warranted.[46]

In conclusion, in two large North American MASLD cohorts assessed in primary care, we show that initial triage with FIB-4 requires refinement for individuals with obesity and diabetes, because a substantial proportion of patients with elevated liver stiffness may be missed. This issue is particularly relevant for selecting MASLD patients for newly available pharmacologic treatments, which are largely based on NITs indicating advanced fibrosis.

Affiliations

[1]*Division of Gastroenterology and Hepatology, Department of Medicine, Cumming School of Medicine, University of Calgary, Calgary, Alberta, Canada;* [2]*Department of Community Health Sciences, Cumming School of Medicine, University of Calgary, Calgary, Alberta, Canada;* [3]*Division of Gastroenterology, Liver Unit, University of Alberta, Edmonton, Alberta, Canada;* [4]*Division of Gastroenterology and Hepatology, McGill University, Montreal, Quebec, Canada;* [5]*Provincial Research Data Services, Health Shared Services, Calgary, Alberta, Canada;* [6]*Department of Radiology, Cumming School of Medicine, University of Calgary, Calgary, Alberta, Canada*

Abbreviations

2D-SWE, two-dimensional shear wave elastography; ALD, alcohol-related liver disease; ALT, alanine aminotransferase; AST, aspartate aminotransferase; FIB-4, fibrosis-4 index; FNI, fibrotic NASH index; HbA1c, hemoglobin A1c; INR, international normalized ratio; LSM, liver stiffness measurement; MASLD, metabolic dysfunction-associated steatotic liver disease; MetALD, metabolic dysfunction- and alcohol-associated liver disease; VCTE, vibration-controlled transient elastography.

Financial support

This work was funded through a Canadian Institutes of Health Research (CIHR) Project Grant and a Gilead Sciences investigator-initiated grant awarded to Dr. A Shaheen.

Conflicts of interest

AAS has participated in advisory boards and received research grants from Gilead, Novo Nordisk and Intercept. JGA received grants from Cook and Gilead (paid to the University of Alberta) and received consulting fees from Boehringer Ingelheim, AstraZeneca, Inventiva and 89Bio.
Please refer to the accompanying ICMJE disclosure forms for further details.

Authors' contributions

AAS, EB and JGA designed the study. AAS, EB, MK, MT, MMM, JJ, FL, WS, AM and JGA collected and analyzed the data. AAS, EB, MS, GS and JGA drafted the manuscript. All Authors interpreted the data and provided critical revisions of the manuscript for important intellectual contents. All authors have approved the final draft of the manuscript.

Data availability

The data supporting the findings of this study are not publicly available because of ethics and privacy restrictions. De-identified data may be shared upon reasonable request and with approval from the University of Calgary Research Ethics Board (ethics committee), and subject to any required institutional data access agreements.

Acknowledgment

We would like to thank Dr. Katrine Prier Lindvig and the Evido team for their support in providing the LiverPRO calculations.

Supplementary data

Supplementary data to this article can be found online at https://doi.org/10.1016/j.jhepr.2026.101735.

References

[1] Riazi K, Azhari H, Charette JH, et al. The prevalence and incidence of NAFLD worldwide: a systematic review and meta-analysis. Lancet Gastroenterol Hepatol 2022;7(9):851–861. Epub 20220705.

[2] Rinella ME, Lazarus JV, Ratziu V, et al. A multi-society Delphi consensus statement on new fatty liver disease nomenclature. Ann Hepatol 2023:101133. Epub 20230620.

[3] Hagstrom H, Nasr P, Ekstedt M, et al. Fibrosis stage but not NASH predicts mortality and time to development of severe liver disease in biopsy-proven NAFLD. J Hepatol 2017;67(6):1265–1273. Epub 2017/08/15.

[4] Mantovani A, Csermely A, Petracca G, et al. Non-alcoholic fatty liver disease and risk of fatal and non-fatal cardiovascular events: an updated systematic review and meta-analysis. Lancet Gastroenterol Hepatol 2021;6(11):903–913. Epub 20210921.

[5] Kanwal F, Shubrook JH, Adams LA, et al. Clinical care pathway for the risk stratification and management of patients with nonalcoholic fatty liver disease. Gastroenterology 2021;161(5):1657–1669. Epub 20210920.

[6] Abeysekera KWM, Macpherson I, Glyn-Owen K, et al. Community pathways for the early detection and risk stratification of chronic liver disease: a narrative systematic review. Lancet Gastroenterol Hepatol 2022;7(8):770–780. Epub 20220505.

[7] Lazarus JV, Anstee QM, Hagstrom H, et al. Defining comprehensive models of care for NAFLD. Nat Rev Gastroenterol Hepatol 2021;18(10):717–729. Epub 20210625.

[8] Tavaglione F, Jamialahmadi O, De Vincentis A, et al. Development and validation of a score for fibrotic nonalcoholic steatohepatitis. Clin Gastroenterol Hepatol 2023;21(6):1523–15232 e1. Epub 20220411.

[9] Noureddin M, Charlton MR, Harrison SA, et al. Expert panel recommendations: practical clinical applications for initiating and monitoring resmetirom

in patients with MASH/NASH and moderate to noncirrhotic advanced fibrosis. Clin Gastroenterol Hepatol 2024;22(12):2367–2377. Epub 20240720.
[10] Chen VL, Morgan TR, Rotman Y, et al. Resmetirom therapy for metabolic dysfunction-associated steatotic liver disease: october 2024 updates to AASLD Practice Guidance. Hepatology 2025;81(1):312–320. Epub 20241018.
[11] European Association for the Study of the Liver, European Association for the Study of D, European Association for the Study of O. EASL-EASD-EASO Clinical Practice Guidelines on the management of metabolic dysfunction-associated steatotic liver disease (MASLD): executive Summary. Diabetologia 2024;67(11):2375–2392.
[12] Kim RG, Deng J, Reaso JN, et al. Noninvasive fibrosis screening in fatty liver disease among vulnerable populations: impact of diabetes and obesity on FIB-4 score accuracy. Diabetes Care 2022;45(10):2449–2451.
[13] Davyduke T, Tandon P, Al-Karaghouli M, et al. Impact of implementing a "FIB-4 first" strategy on a pathway for patients with NAFLD referred from primary care. Hepatol Commun 2019;3(10):1322–1333. Epub 20190729.
[14] Shaheen AA, Riazi K, Medellin A, et al. Risk stratification of patients with nonalcoholic fatty liver disease using a case identification pathway in primary care: a cross-sectional study. CMAJ Open 2020;8(2):E370–E376. Epub 20200515.
[15] Sung S, Al-Karaghouli M, Tam M, et al. Age-dependent differences in FIB-4 predictions of fibrosis in patients with MASLD referred from primary care. Hepatol Commun 2025;9(1). Epub 20241211.
[16] Kim DW, Suh CH, Kim KW, et al. Technical performance of two-dimensional shear wave elastography for measuring liver stiffness: a systematic review and meta-analysis. Korean J Radiol 2019;20(6):880–893. Epub 2019/05/28.
[17] Kim HC, Nam CM, Jee SH, et al. Normal serum aminotransferase concentration and risk of mortality from liver diseases: prospective cohort study. BMJ 2004;328(7446):983. Epub 20040317.
[18] Lindvig KP, Thorhauge KH, Hansen JK, et al. Development, validation, and prognostic evaluation of LiverPRO for the prediction of significant liver fibrosis in primary care: a prospective cohort study. Lancet Gastroenterol Hepatol 2025;10(1):55–67.
[19] DeLong ER, DeLong DM, Clarke-Pearson DL. Comparing the areas under two or more correlated receiver operating characteristic curves: a nonparametric approach. Biometrics 1988;44(3):837–845. Epub 1988/09/01.
[20] Quan H, Li B, Couris CM, et al. Updating and validating the Charlson comorbidity index and score for risk adjustment in hospital discharge abstracts using data from 6 countries. Am J Epidemiol 2011;173(6):676–682. Epub 2011/02/19.
[21] Liu Q, Shepherd BE, Li C, et al. Modeling continuous response variables using ordinal regression. Stat Med 2017;36(27):4316–4335. Epub 20170905.
[22] Barr RG, Wilson SR, Rubens D, et al. Update to the society of radiologists in ultrasound liver elastography consensus statement. Radiology 2020;296(2):263–274. Epub 20200609.
[23] Ho D, Imai K, King G, et al. MatchIt: nonparametric preprocessing for parametric causal inference. J Stat Softw 2011;42(8):1–28.
[24] Kim D, Konyn P, Sandhu KK, et al. Metabolic dysfunction-associated fatty liver disease is associated with increased all-cause mortality in the United States. J Hepatol 2021;75(6):1284–1291. Epub 20210808.
[25] Zhang X, Heredia NI, Balakrishnan M, et al. Prevalence and factors associated with NAFLD detected by vibration controlled transient elastography among US adults: results from NHANES 2017-2018. PLoS One 2021;16(6): e0252164. Epub 2021/06/04.
[26] Yip TC, Lee HW, Lin H, et al. Prognostic performance of the two-step clinical care pathway in metabolic dysfunction-associated steatotic liver disease. J Hepatol 2025;83:304–314.
[27] Srivastava A, Gailer R, Tanwar S, et al. Prospective evaluation of a primary care referral pathway for patients with non-alcoholic fatty liver disease. J Hepatol 2019;71(2):371–378. Epub 20190406.
[28] Macpherson I, Nobes JH, Dow E, et al. Intelligent liver function testing: working smarter to improve patient outcomes in liver disease. J Appl Lab Med 2020;5(5):1090–1100.
[29] El-Gohary M, Moore M, Roderick P, et al. Local care and treatment of liver disease (LOCATE) - a cluster-randomized feasibility study to discover, assess and manage early liver disease in primary care. PLoS One 2018;13(12):e0208798. Epub 20181221.
[30] Mansour D, Grapes A, Herscovitz M, et al. Embedding assessment of liver fibrosis into routine diabetic review in primary care. JHEP Rep 2021;3(4): 100293. Epub 20210422.
[31] Hayward KL, McKillen BJ, Horsfall LU, et al. Towards collaborative management of non-alcoholic fatty liver disease: a 'real-world' pathway for fibrosis risk assessment in primary care. Intern Med J 2022;52(10):1749–1758. Epub 20220607.
[32] Gines P, Castera L, Lammert F, et al. Population screening for liver fibrosis: toward early diagnosis and intervention for chronic liver diseases. Hepatology 2022;75(1):219–228. Epub 20211210.
[33] Harris R, Card TR, Delahooke T, et al. Obesity is the most common risk factor for chronic liver disease: results from a risk stratification pathway using transient elastography. Am J Gastroenterol 2019;114(11):1744–1752.
[34] Pierce TT, Ozturk A, Sherlock SP, et al. Reproducibility and repeatability of US shear-wave and transient elastography in nonalcoholic fatty liver disease. Radiology 2024;312(3):e233094.
[35] Balakrishnan M, Patel P, Dunn-Valadez S, et al. Women have a lower risk of nonalcoholic fatty liver disease but a higher risk of progression vs men: a systematic review and meta-analysis. Clin Gastroenterol Hepatol 2021;19(1):61–71 e15. Epub 20200430.
[36] Qadri S, Ahlholm N, Lonsmann I, et al. Obesity modifies the performance of fibrosis biomarkers in nonalcoholic fatty liver disease. J Clin Endocrinol Metab 2022;107(5):e2008–e2020.
[37] European Association for the Study of the Liver. EASL Clinical Practice Guidelines on non-invasive tests for evaluation of liver disease severity and prognosis - 2021 update. J Hepatol 2021;75(3):659–689. Epub 20210621.
[38] Hirose S, Matsumoto K, Tatemichi M, et al. Nineteen-year prognosis in Japanese patients with biopsy-proven nonalcoholic fatty liver disease: lean versus overweight patients. PLoS One 2020;15(11):e0241770. Epub 20201113.
[39] Drolz A, Wolter S, Wehmeyer MH, et al. Performance of non-invasive fibrosis scores in non-alcoholic fatty liver disease with and without morbid obesity. Int J Obes (Lond) 2021;45(10):2197–2204. Epub 20210624.
[40] Quek J, Chan KE, Wong ZY, et al. Global prevalence of non-alcoholic fatty liver disease and non-alcoholic steatohepatitis in the overweight and obese population: a systematic review and meta-analysis. Lancet Gastroenterol Hepatol 2023;8(1):20–30. Epub 20221116.
[41] Chouik Y, Aubin A, Maynard-Muet M, et al. The grade of obesity affects the noninvasive diagnosis of advanced fibrosis in individuals with MASLD. Obesity (Silver Spring) 2024;32(6):1114–1124. Epub 20240503.
[42] Jarvis H, Craig D, Barker R, et al. Metabolic risk factors and incident advanced liver disease in non-alcoholic fatty liver disease (NAFLD): a systematic review and meta-analysis of population-based observational studies. Plos Med 2020;17(4):e1003100. Epub 20200430.
[43] Park H, Cheuk-Fung Yip T, Yoon EL, et al. Impact of cardiometabolic risk factors on hepatic fibrosis and clinical outcomes in MASLD: a population-based multi-cohort study. JHEP Rep 2025;7(6):101388. Epub 20250307.
[44] Blank V, Petroff D, Beer S, et al. Current NAFLD guidelines for risk stratification in diabetic patients have poor diagnostic discrimination. Sci Rep 2020;10(1):18345. Epub 20201027.
[45] Udompap P, Therneau TM, Canning RE, et al. Performance of American Gastroenterological Association Clinical Care Pathway for the risk stratification of patients with nonalcoholic fatty liver disease in the US population. Hepatology 2023;77(3):931–941. Epub 20230217.
[46] Congly SE, Shaheen AA, Swain MG. Modelling the cost effectiveness of non-alcoholic fatty liver disease risk stratification strategies in the community setting. PLoS One 2021;16(5):e0251741. Epub 2021/05/22.

Keywords: MASLD; Metabolic dysfunction-associated steatotic liver disease; risk-stratification; primary care; advanced fibrosis.

Received 24 April 2025; received in revised form 18 December 2025; accepted 30 December 2025; Available online 12 January 2026

Journal of Hepatology, Volume *8*

Supplemental information

Diabetes and obesity reduce FIB-4 accuracy in MASLD referral pathways

Abdel-Aziz Shaheen, Elizabeth Baguley, Mark G. Swain, Matthew Tam, Mang Ming Ma, Giada Sebastiani, Jason Jiang, Frank Lee, Alexandra Medellin, and Juan G. Abraldes

Diabetes and obesity reduce FIB-4 accuracy in MASLD referral pathways

Abdel-Aziz Shaheen, Elizabeth Baguley, Mark G Swain, Matthew Tam, Mang Ming Ma, Giada Sebastiani, Jason Jiang, Frank Lee, Alexandra Medellin, Juan G Abraldes

Table of contents

Model building process……………………………………………………………………2

Results of the matching process…………………………………………………………4

Fig. S1……………………………………………………………………………………5

Table S1…………………………………………………………………………………6

Table S2…………………………………………………………………………………7

Table S3…………………………………………………………………………………8

Table S4…………………………………………………………………………………9

Table S5………………………………………………………………………………...10

Table S6………………………………………………………………………………...11

Table S7………………………………………………………………………………...12

Model building process

The modeling process, based on ordinal regression for a continuous outcome, was conducted following methods and code described in https://hbiostat.org/rmsc/cony#ordinal-regression-models-for-continuous-y.

The goal of the modeling process was to assess how the variables of interest contribute to the prediction of SWE, which is primarily a descriptive modeling question. The log-log link was chosen since it provided a better fit to the data than the logit link. FIB-4 was modeled with restricted cubic splines (4 knots). To show the effects of these variables on SWE predictions we plotted the exceedance probabilities of different values of SWE for the commonly used FIB-4 threshold values of 1.3 and 2.67.

To develop a unified model to predict different SWE thresholds according to FIB-4 and the 5 variables of interest we used a similar approach. After backwards elimination (with AIC as stopping rule, i.e., (i.e., a factor is deleted if the $\chi 2$ falls below twice its degrees of freedom) FIB-4, BMI (both modeled with restricted cubic splines with 4 knots), sex, diabetes and abnormal transaminases were retained in the model.

The table shows the relative explained variation (with 95% CI) of each variable to explain the variability in SWE

Relative Explained Variation (REV)

	REV	Lower CI	Upper CI
FIB4	0.435	0.377	0.504
Sex	0.013	0.005	0.028
BMI	0.319	0.282	0.375
diabetes	0.118	0.071	0.145
abnormal ALT	0.029	0.011	0.046

The nomogram below provides a graphical representation of the model. The nomogram translates the multivariable ordinal regression model into a point-based tool that allows to grasp the relative contribution of each variable to the model. To use the nomogram: (1) For each predictor (FIB-4, BMI, sex, diabetes, abnormal ALT), locate the patient's value on its axis. (2) Draw a vertical line to the "Points" axis to assign points for that predictor. (3) Sum points across all predictors to obtain the "Total Points." (4) Locate the "Total Points" on the corresponding axis and draw a vertical line downward to read the exceedance probabilities for each threshold of SWE (7,8,10,13 and 17 kPa are shown as examples) on the bottom scales. For example, a patient with a FIB4 of 1.3 (˜41 points), Male (˜10 points), with a BMI of 35 (˜34 points), with diabetes (˜33 points) and abnormal ALT (˜15 points), would have a total of ˜133 points. That would give the patient a 25% chances *(exceedance probability*) of having a SWE of 7 kPa or more , a 19% of 8 kPa or more , and 11% of 10 kPa or more, a 5% of 13 kPa or more and a 2% of having 17 kPa or more.

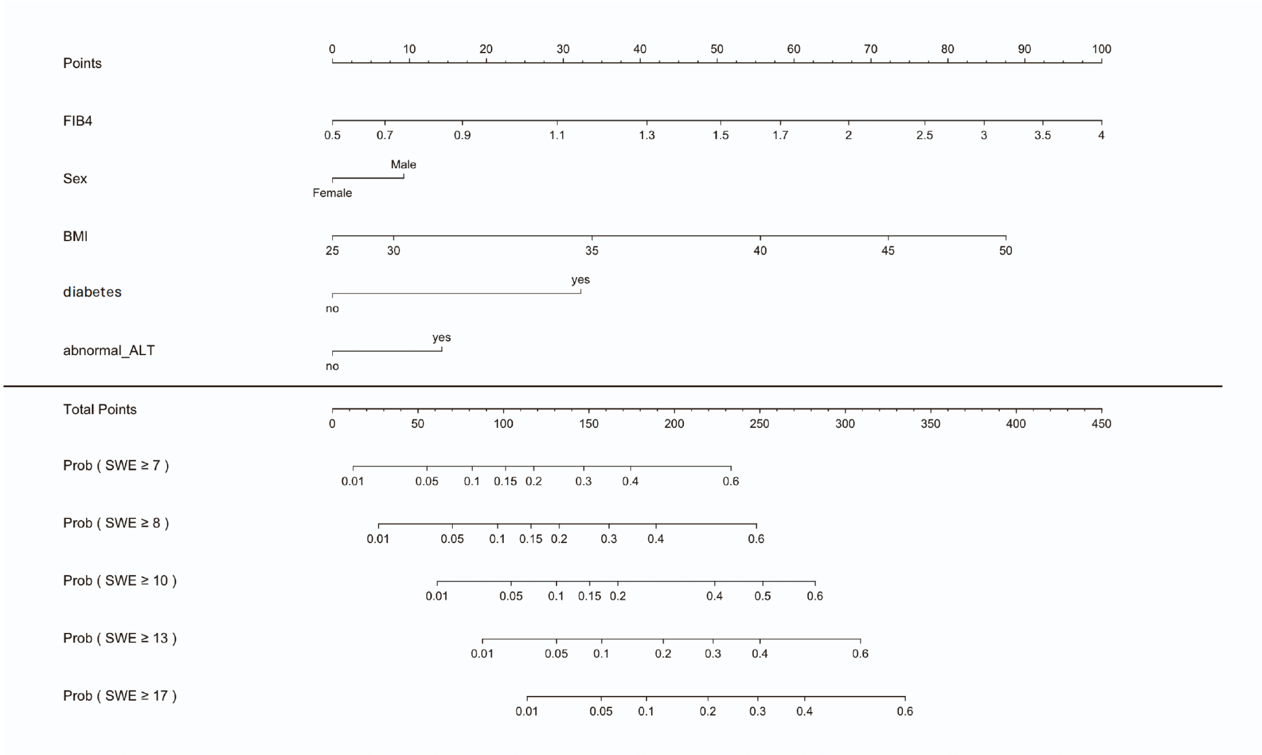

The formula to calculate the exact exceedance probabilities for a given SWE threshold is as follows:

Exceedance probability of having (SWE>=Y) = exp(-exp(-linear_predictor))

Where the linear_predictor is calculated as follows

```
Linear_predictor = Intercept (specific for every SWE value) +
        0.2603354 * FIB4 +
        0.3864207 * max(FIB4 - 0.38199998, 0)^3 -
        0.94166768 * max(FIB4 - 0.78029412, 0)^3 +
        0.61079383 * max(FIB4 - 1.2038862, 0)^3 -
        0.055546848 * max(FIB4 - 2.6673195, 0)^3 +
        0.099700558 * (Sex == "Male") +
        0.0068417906 * BMI +
        0.00027493356 * max(BMI - 23.23, 0)^3 -
        0.00074095161 * max(BMI - 29.04, 0)^3 +
        0.00051799554 * max(BMI - 33.66, 0)^3 -
        5.1977492e-05 * max(BMI - 44.35, 0)^3 +
        0.31463483 * (diabetes == "yes") +
```

0.16062209 * (abnormal ALT == "yes")

Intercepts for selected thresholds of SWE

y>=6.5	-1.8059
y>=7	-1.9485
y>=8	-2.0948
y>=9	-2.2944
y>=10	-2.4183
y>=11	-2.5179
y>=12	-2.5985
y>=13	-2.6786
y>=14	-2.7401
y>=15	-2.7940
y>=16	-2.8537
y>=17	-2.9323

Results of the matching process

Matching between Edmonton and Calgary cohorts were performed as described in methods in the main manuscript.
The different characteristics of the two cohorts are shown in table 1
The following table show the descriptives of the sample after matching

Post Matching

	Means Edmonton	Means Calgary	Std. Mean Diff.
diabetes	0.2213	0.2122	0.0220
BMI	31.5960	31.6129	-0.0030
abnormal_ALT	0.9147	0.9025	0.0436
age	44.1878	43.9513	0.0185
FemaleSex	0.3259	0.3239	0.0043

Fig. S1: Inclusion and exclusion criteria for the Calgary and Edmonton MASLD cohorts

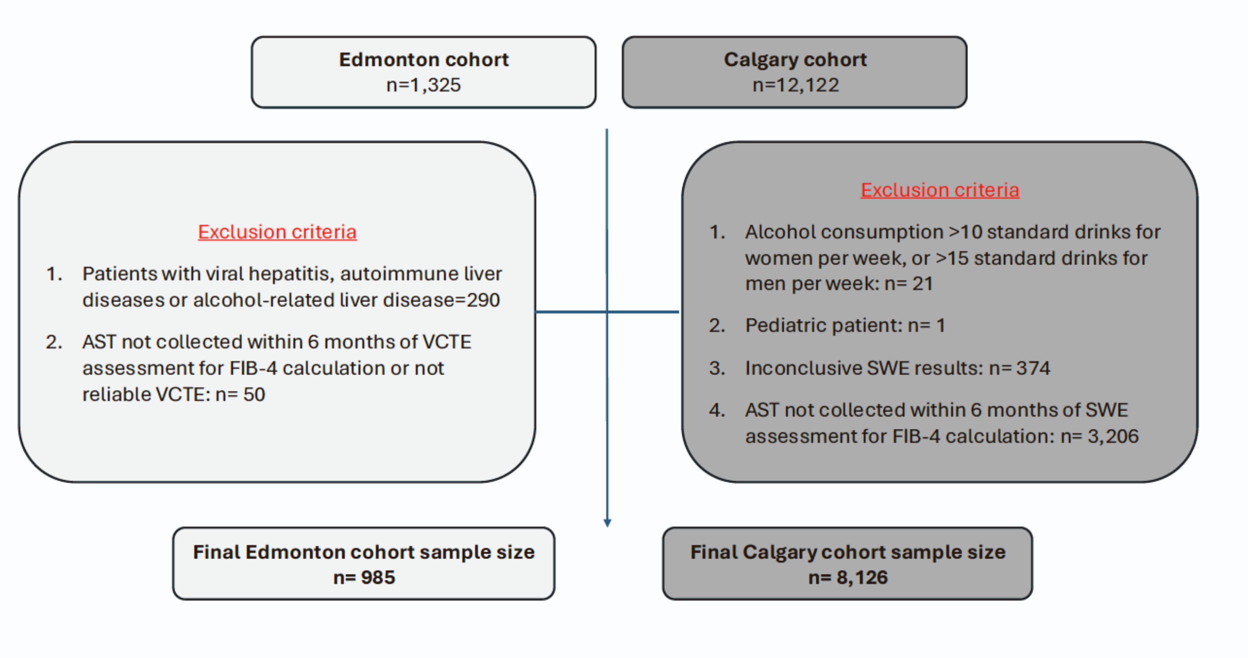

Table S1: Comparison of patient characteristics of the Calgary MASLD pathway according to FIB-4 availability

Characteristic	Calgary MASLD cohort patients with FIB-4 N=8,126	Calgary MASLD cohort patients without FIB-4 N=3,532	P value
Age, yrs.	54 (43-63)	55 (44-63)	0.125
Female sex	52.5% (4,261)	49.2% (1,732)	0.001
BMI (Kg/Height in meter^2)	31.2 (27.6-35.7)	31.5 (27.9-35.9)	0.070
Baseline investigations			
Albumin, g/L	39 (37-41)	39 (37-41)	0.043
Alkaline Phosphatase, U/L	77 (64-95)	76 (63-93)	<0.001
Triglycerides, mmol/L	1.8 (1.2-2.5)	1.7 (1.3-2.5)	0.522
HDL, mmol/L	1.2 (1.0-1.4)	1.2 (1.0-1.4)	0.151
LDL, mmol/L	2.6 (1.9-3.3)	2.7 (2.0-3.3)	0.002
Total bilirubin, μmol/L	8 (6-11)	8 (6-11)	0.089
HbA1c, %	5.8 (5.5-6.2)	5.8 (5.5-6.2)	0.086
Diabetes mellitus	34.1% (2,772)	31.9% (1,128)	0.022
Comorbidities, Charlson Index			
0	34.4% (2,791)	39.6% (1,398)	
1	21.8% (1,769)	21.8% (771)	<0.001
≥ 2	43.9% (3,566)	38.6% (1,363)	
SWE, valid measurements in kPa	4.6 (3.8-5.7)	4.5 (3.8-5.4)	<0.001

Data presented as median (IQR) or % (n). BMI, body mass index; ALT, alanine aminotransferase; AST, aspartate aminotransferase; HDL, high density lipoprotein; LDL, low density lipoprotein; HbA1C, Hemoglobin A1C; SWE, shearwave elastography; FIB-4, fibrosis 4 variable score.

Table S2: Characteristics of the Calgary MASLD clinic patients according to sex.

Characteristic	Female patients<br>N=4,261, 52.5%	Male patients<br>N=3,852, 47.5%	P value
Age, yrs.	55 (46-63)	52 (41-62)	<0.001
BMI (Kg/Height in meter^2)	31.6 (27.4-36.7)	30.9 (27.8-34.7)	<0.001
Baseline investigations			
ALT, U/L	34 (22-53)	43 (28-68)	<0.001
AST, U/L	27 (20-38)	29 (22-41)	<0.001
Albumin, g/L	38 (36-41)	40 (38-42)	<0.001
Alkaline Phosphatase, U/L	82 (68-101)	73 (61-88)	<0.001
Platelets, 10E^9/L	269 (229-315)	232 (196-273)	<0.001
Triglycerides, mmol/L	1.7 (1.2-2.4)	1.8 (1.3-2.7)	<0.001
HDL, mmol/L	1.3 (1.1-1.5)	1.1 (0.9-1.2)	<0.001
LDL, mmol/L	2.7 (2.0-3.3)	2.5 (1.8-3.2)	<0.001
Total bilirubin, μmol/L	7 (5-10)	10 (7-13)	<0.001
HbA1C, %	5.8 (5.5-6.2)	5.7 (5.5-6.2)	<0.001
Diabetes mellitus	35.6% (1,516)	32.5% (1,251)	0.003
Comorbidities, Charlson Index			
0	30.2% (1,286)	38.9% (1,499)	<0.001
1	22.5% (958)	21.0% (810)	
≥ 2	47.3% (2,017)	40.1% (1,543)	
SWE, valid measurements in kPa	4.5 (3.8-5.7)	4.7 (3.9-5.8)	<0.001
SWE ≥ 8 kPa	9.4% (402)	9.1% (350)	0.589
FIB-4	0.95 (0.66-1.37)	0.99 (0.68-1.47)	<0.001
FIB-4 ≥ 1.30	28.0% (1,191)	32.7% (1,261)	<0.001
FIB-4 ≥ 2.65	4.9% (209)	5.3% (205)	0.394

Data presented as median (IQR) or % (n). BMI, body mass index; ALT, alanine aminotransferase; AST, aspartate aminotransferase; HDL, high density lipoprotein; LDL, low density lipoprotein; HbA1C, Hemoglobin A1C; SWE, shearwave elastography; FIB-4, fibrosis 4 variable score.

Table S3: Patients characteristics of the Calgary MASLD clinic according to normality of ALT at baseline.

Characteristic	Patients with normal ALT<br><br>N=2,406, 29.6%	Patients with elevated ALT<br><br>N=5,720, 70.4%	P value
Age, yrs.	57 (46-66)	53 (42-61)	<0.001
Female sex	55.8% (1,335)	51.2% (2,926)	<0.001
BMI (Kg/Height in meter^2)	30.9 (26.9-35.6)	31.4 (27.8-35.7)	0.008
Baseline investigations			
ALT, U/L	20 (16-23)	50 (36-71)	<0.001
AST, U/L	20 (17-24)	33 (25-46)	<0.001
Albumin, g/L	38 (36-41)	40 (37-42)	<0.001
Alkaline Phosphatase, U/L	74 (62-91)	79 (65-97)	<0.001
Platelets, 10E^9/L	256 (212-301)	249 (209-295)	<0.001
Triglycerides, mmol/L	1.6 (1.1-2.3)	1.8 (1.3-2.6)	<0.001
HDL, mmol/L	1.2 (1.0-1.4)	1.1 (1.0-1.4)	<0.001
LDL, mmol/L	2.5 (1.9-3.2)	2.6 (2.0-3.3)	<0.001
Total bilirubin, μmol/L	8 (5-11)	8 (6-12)	<0.001
HbA1C, %	5.7 (5.4-6.1)	5.8 (5.5-6.2)	<0.001
Diabetes mellitus	33.2% (798)	34.5% (1,974)	0.244
Comorbidities, Charlson Index			
0	31.1% (749)	35.7% (2,042)	
1	23.1% (556)	21.2% (1,213)	<0.001
≥ 2	45.8% (1,101)	43.1% (2,465)	
SWE, valid measurements in kPa	4.4 (3.7-5.5)	4.7 (3.9-5.8)	<0.001
SWE ≥ 8 kPa	8.4% (203)	9.6% (551)	0.090
FIB-4	1.00 (0.70-1.41)	0.95 (0.66-1.42)	0.007
FIB-4 ≥ 1.30	30.5% (733)	30.1% (1,723)	0.758
FIB-4 ≥ 2.67	3.5% (85)	5.8% (329)	<0.001

Data presented as median (IQR) or % (n). BMI, body mass index; ALT, alanine aminotransferase; AST, aspartate aminotransferase; HDL, high density lipoprotein; LDL, low density lipoprotein; HbA1C, Hemoglobin A1C; SWE, shearwave elastography; FIB-4, fibrosis 4 variable score.

Table S4: Characteristics of the Calgary MASLD clinic patients according to body mass index (BMI) cutoff 35.

Characteristic	Patients with BMI <35<br>N=4,699, 71.8%	Patients with BMI ≥35<br>N=1,845, 28.2%	P value
Age, yrs.	55 (44-64)	52 (42-61)	<0.001
Female sex	50.1% (2,351)	61.0% (1,124)	<0.001
Baseline investigations			
ALT, U/L	38 (25-60)	38 (25-60)	0.733
AST, U/L	28 (21-39)	27 (20-40)	0.155
Albumin, g/L	40 (38-42)	38 (36-41)	<0.001
Alkaline Phosphatase, U/L	76 (63-94)	79 (65-96)	<0.001
Platelets, $10E^9$/L	250 (209-295)	254 (215-305)	0.002
Triglycerides, mmol/L	1.8 (1.2-2.5)	1.8 (1.3-2.5)	0.001
HDL, mmol/L	1.2 (1.0-1.4)	1.1 (0.9-1.3)	<0.001
LDL, mmol/L	2.7 (2.0-3.3)	2.5 (1.9-3.2)	<0.001
Total bilirubin, μmol/L	8 (6-12)	8 (6-11)	<0.001
HbA1C, %	5.7 (5.5-6.1)	5.9 (5.5-6.4)	<0.001
Diabetes mellitus	30.3% (1,423)	42.2% (778)	<0.001
Comorbidities, Charlson Index			
0	36.9% (1,733)	28.0% (517)	
1	22.4% (1,053)	19.5% (359)	<0.001
≥ 2	40.7% (1,913)	52.5% (969)	
SWE, valid measurements in kPa	4.4 (3.7-5.4)	5.1 (4.2-6.4)	<0.001
SWE ≥ 8 kPa	6.4% (301)	15.2% (280)	<0.001
FIB-4	0.98 (0.69-1.43)	0.92 (0.63-1.36)	<0.001
FIB-4 ≥ 1.30	30.8% (1,446)	27.4% (506)	0.008
FIB-4 ≥ 2.67	5.4% (253)	4.1% (76)	0.035

Data presented as median (IQR) or % (n). BMI, body mass index; ALT, alanine aminotransferase; AST, aspartate aminotransferase; HDL, high density lipoprotein; LDL, low density lipoprotein; HbA1C, Hemoglobin A1C; SWE, shearwave elastography; FIB-4, fibrosis 4 variable score.

Table S5: Characteristics of the Calgary MASLD clinic patients according to body mass index (BMI) cutoff 30.

Characteristic	Patients with BMI <30<br>N=2,718, 41.6%	Patients with BMI ≥30<br>N=3,824, 58.5%	P value
Age, yrs.	55 (45-64)	54 (43-62)	<0.001
Female sex	51.4% (1,393)	54.5% (2,080)	0.012
Baseline investigations			
ALT, U/L	37 (24-58)	39 (25-61)	0.003
AST, U/L	28 (21-38)	28 (21-40)	0.955
Albumin, g/L	40 (38-42)	39 (37-41)	<0.001
Alkaline Phosphatase, U/L	76 (63-93)	78 (65-95)	0.001
Platelets, $10E^9/L$	249 (209-296)	253 (211-299)	0.022
Triglycerides, mmol/L	1.7 (1.2-2.4)	1.8 (1.3-2.6)	<0.001
HDL, mmol/L	1.2 (1.0-1.5)	1.1 (0.9-1.3)	<0.001
LDL, mmol/L	2.7 (2.0-3.3)	2.6 (1.9-3.2)	<0.001
Total bilirubin, μmol/L	9 (6-12)	8 (6-11)	<0.001
HbA1C, %	5.7 (5.4-6.1)	5.8 (5.5-6.3)	<0.001
Diabetes mellitus	28.6% (777)	37.2% (1,423)	<0.001
Comorbidities, Charlson Index			
0	38.6% (1,050)	31.4% (1,199)	
1	22.4% (608)	21.0% (804)	<0.001
≥ 2	39.0% (1,060)	47.6% (1,821)	
SWE, valid measurements in kPa	4.3 (3.7-5.2)	4.8 (4.0-6.0)	<0.001
SWE ≥ 8 kPa	5.5% (150)	11.3% (431)	<0.001
FIB-4	1.00 (0.69-1.45)	0.94 (0.65-1.38)	<0.001
FIB-4 ≥ 1.30	31.4% (853)	28.7% (1,099)	0.021
FIB-4 ≥ 2.67	5.6% (152)	4.6% (177)	0.079

Data presented as median (IQR) or % (n). BMI, body mass index; ALT, alanine aminotransferase; AST, aspartate aminotransferase; HDL, high density lipoprotein; LDL, low density lipoprotein; HbA1C, Hemoglobin A1C; SWE, shearwave elastography; FIB-4, fibrosis 4 variable score.

Table S6: Characteristics of the Calgary MASLD clinic patients according to having diabetes mellitus.

Characteristic	Patients without diabetes<br><br>N=5,354 , 65.9%	Patients with diabetes<br><br>N=2,772, 34.1%	P value
Age, yrs.	52 (41-61)	58 (48-66)	<0.001
Female sex	51.4% (2,754)	54.8% (1,516)	0.003
BMI (Kg/Height in meter^2)	30.7 (27.3-34.8)	32.3 (28.3-37.5)	<0.001
Baseline investigations			
ALT, U/L	38 (24-61)	39 (25-60)	0.387
AST, U/L	28 (21-39)	28 (21-41)	0.101
Albumin, g/L	39 (37-42)	39 (37-41)	<0.001
Alkaline Phosphatase, U/L	77 (64-95)	78 (65-96)	0.015
Platelets, 10E^9/L	254 (214-297)	248 (203-297)	<0.001
Triglycerides, mmol/L	1.7 (1.2-2.4)	1.9 (1.3-2.6)	<0.001
HDL, mmol/L	1.2 (1.0-1.4)	1.1 (0.9-1.3)	<0.001
LDL, mmol/L	2.8 (2.2-3.4)	2.2 (1.5-2.9)	<0.001
Total bilirubin, µmol/L	8 (6-12)	8 (6-11)	0.217
HbA1C, %	5.6 (5.4-5.8)	6.6 (6.1-7.4)	<0.001
Comorbidities, Charlson Index<br>0<br>1<br>≥ 2	 50.0% (2,675)<br>31.6% (1,694)<br>18.4% (985)	 4.2% (116)<br>2.7% (75)<br>93.1% (2,581)	<0.001
SWE, valid measurements in kPa<br>SWE ≥ 8 kPa	4.4 (3.7-5.4)<br>6.0% (320)	5.1 (4.2-6.4)<br>15.7% (434)	<0.001<br><0.001
FIB-4<br>FIB-4 ≥ 1.30<br>FIB-4 ≥ 2.67	0.91 (0.64-1.34)<br>26.4% (1,414)<br>3.9% (209)	1.07 (0.74-1.58)<br>37.6 % (1,042)<br>7.4% (205)	<0.001<br><0.001<br><0.001

Data presented as median (IQR) or % (n). BMI, body mass index; ALT, alanine aminotransferase; AST, aspartate aminotransferase; HDL, high density lipoprotein; LDL, low density lipoprotein; HbA1C, Hemoglobin A1C; SWE, shearwave elastography; FIB-4, fibrosis 4 variable score.

Table S7: Characteristics of the Calgary MASLD clinic patients according to age ≥65

Characteristic	Patients ≥ 65 years<br>N=1,675, 20.6%	Patients <65 years<br>N=6,451 , 79.4%	P value
Female sex	52.8% (884)	52.5% (3,377)	0.792
BMI (Kg/Height in meter^2)	30.6 (27.2-34.6)	31.4 (27.6-36.0)	<0.001
Baseline investigations			
ALT, U/L	31 (21-46)	41 (26-64)	<0.001
AST, U/L	26 (20-37)	28 (21-40)	<0.001
Albumin, g/L	38 (36-40)	39 (37-42)	<0.001
Alkaline Phosphatase, U/L	78 (64-96)	77 (64-95)	0.831
Platelets, 10E^9/L	228 (187-271)	257 (217-302)	<0.001
Triglycerides, mmol/L	1.6 (1.2-2.3)	1.8 (1.3-2.6)	<0.001
HDL, mmol/L	1.2 (1.0-1.5)	1.1 (1.0-1.4)	<0.001
LDL, mmol/L	2.2 (1.6-2.9)	2.7 (2.1-3.3)	<0.001
Total bilirubin, μmol/L	9 (6-12)	8 (6-11)	<0.001
HbA1C, %	6.0 (5.6-6.6)	5.7 (5.4-6.1)	<0.001
Diabetes mellitus	47.8% (800)	30.6% (1,972)	<0.001
Comorbidities, Charlson Index			
0	18.8% (314)	38.4% (2,477)	<0.001
1	17.9% (299)	22.8% (1,470)	
≥ 2	63.4% (1,062)	38.8% (2,504)	
SWE, valid measurements in kPa	4.9 (4.1-6.3)	4.5 (3.8-5.6)	<0.001
SWE ≥ 8 kPa	14.9% (249)	7.8% (505)	<0.001
FIB-4	1.51 (1.15-2.08)	0.86 (0.61-1.21)	<0.001
FIB-4 ≥ 1.30	65.3% (1,093)	21.1% (1,363)	<0.001
FIB-4 ≥ 2.65	13.4% (225)	2.9% (189)	<0.001

Data presented as median (IQR) or % (n). BMI, body mass index; ALT, alanine aminotransferase; AST, aspartate aminotransferase; HDL, high density lipoprotein; LDL, low density lipoprotein; HbA1C, Hemoglobin A1C; SWE, shearwave elastography; FIB-4, fibrosis 4 variable score.
